# Opportunities and challenges of alpha-synuclein as a potential biomarker for Parkinson’s disease and other synucleinopathies

**DOI:** 10.1038/s41531-022-00357-0

**Published:** 2022-07-22

**Authors:** Pedro Magalhães, Hilal A. Lashuel

**Affiliations:** Laboratory of Molecular and Chemical Biology of Neurodegeneration, School of Life Sciences, Brain Mind Institute, École Polytechnique Fédérale de Lausanne (EPFL), CH-1015 Lausanne, Switzerland

**Keywords:** Parkinson's disease, Neuroscience

## Abstract

Parkinson’s disease (PD), the second most common progressive neurodegenerative disease, develops and progresses for 10–15 years before the clinical diagnostic symptoms of the disease are manifested. Furthermore, several aspects of PD pathology overlap with other neurodegenerative diseases (NDDs) linked to alpha-synuclein (aSyn) aggregation, also called synucleinopathies. Therefore, there is an urgent need to discover and validate early diagnostic and prognostic markers that reflect disease pathophysiology, progression, severity, and potential differences in disease mechanisms between PD and other NDDs. The close association between aSyn and the development of pathology in synucleinopathies, along with the identification of aSyn species in biological fluids, has led to increasing interest in aSyn species as potential biomarkers for early diagnosis of PD and differentiate it from other synucleinopathies. In this review, we (1) provide an overview of the progress toward mapping the distribution of aSyn species in the brain, peripheral tissues, and biological fluids; (2) present comparative and critical analysis of previous studies that measured total aSyn as well as other species such as modified and aggregated forms of aSyn in different biological fluids; and (3) highlight conceptual and technical gaps and challenges that could hinder the development and validation of reliable aSyn biomarkers; and (4) outline a series of recommendations to address these challenges. Finally, we propose a combined biomarker approach based on integrating biochemical, aggregation and structure features of aSyn, in addition to other biomarkers of neurodegeneration. We believe that capturing the diversity of aSyn species is essential to develop robust assays and diagnostics for early detection, patient stratification, monitoring of disease progression, and differentiation between synucleinopathies. This could transform clinical trial design and implementation, accelerate the development of new therapies, and improve clinical decisions and treatment strategies.

## Introduction

Parkinson’s disease (PD) is one of the most progressive neurodegenerative diseases (NDDs), with a worldwide prevalence rate of ~1–4% in people aged over 60 years^1^. The incidence of PD is expected to increase as a result of higher life expectancy^2^. PD is characterized by the progressive loss of dopaminergic neurons and the deposition of aggregated alpha-synuclein (aSyn) into intracellular inclusions that accumulate in the form of Lewy bodies (LBs) in cell bodies and Lewy neurites (LNs) in axons and dendrites^3^. To date, clinical PD diagnosis has been based on motor features, along with nonmotor symptoms such as psychiatric and autonomic features and sleep disturbance^4–7^. Detection of aSyn pathology in the postmortem brain remains the primary means of reaching a conclusive diagnosis, often revealing that significant cases of PD have been misdiagnosed^8^. Because the diagnosis of PD relies on clinical symptoms that are manifested only after a substantial and irreversible loss of dopaminergic neurons in the substantia nigra (SN), there is an urgent need to identify PD-specific biomarkers that allow diagnosis at the onset and/or early stages of the disease^9^. Furthermore, given the clinical and neuropathological overlap between PD and other synucleinopathies (e.g., dementia with Lewy bodies (DLB) and multiple system atrophy (MSA)), there is also a need for biomarkers that would allow differentiation between synucleinopathies. The discovery of early diagnostic and prognostic markers that reflect disease pathophysiology, progression and severity and reflect potential differences in disease mechanisms are of paramount importance and hold great promise for improving the design of clinical trials and the development of novel disease-specific diagnostic tools and therapies for PD and other synucleinopathies.

### aSyn as a potential biomarker

Several experimental observations have led to the emergence of aSyn as a leading therapeutic target and biomarker for PD. First, aggregated forms of aSyn are major components of LBs and LNs, the pathological hallmarks of PD^3,10–14^. aSyn aggregates are also found in the brains of patients with other synucleinopathies, including DLB, MSA and Alzheimer’s disease (AD)^15–17^. Second, mutations or duplications of the SNCA gene appear to be sufficient to cause PD or Lewy body dementia (LBD)^18–27^. Several familial forms of PD have been linked to increased expression of aSyn due to SNCA gene duplication or missense point mutations (single amino acid substitutions), such as A30P, E46K, H50Q, A53E and A53T^23,25,26,28–30^. Moreover, other aSyn point mutations, such as the G51D mutation, were reported to phenotypically display common neuropathological features of PD and MSA^31,32^. More recently, an E83Q mutation in aSyn was identified in a patient suffering from DLB and atypical frontotemporal lobar degeneration^27,33^. Third, the level of aSyn aggregates in the cerebrospinal fluid (CSF) and skin biopsies distinguishes PD patients from controls with high accuracy^34–36^. Fourth, several animal models show that overexpression of aSyn (the wild-type form or disease-associated mutant forms) or inoculation of aSyn into the central nervous system (CNS) and peripheral tissues induces aSyn pathology formation and/or pathology spreading into brain regions that are affected in PD and other synucleinopathies^37–39^. These observations, combined with the findings that aSyn aggregation and pathology spreading in rodent models increases with increasing aSyn levels, point to aSyn as a central player in PD pathogenesis^40–43^. However, whether aSyn aggregation and neurodegeneration are the primary initiators of the pathological process in PD remains a subject of active debate. Although several aSyn-targeting therapies are being tested in the clinic, reliable tools and assays to assess aSyn target engagement are still lacking. This has led to increasing efforts to develop and validate assays to identify, quantify and validate different aSyn species as potential biomarkers for synucleinopathies.

### aSyn in body fluids and peripheral tissues

**Fig. 1:**
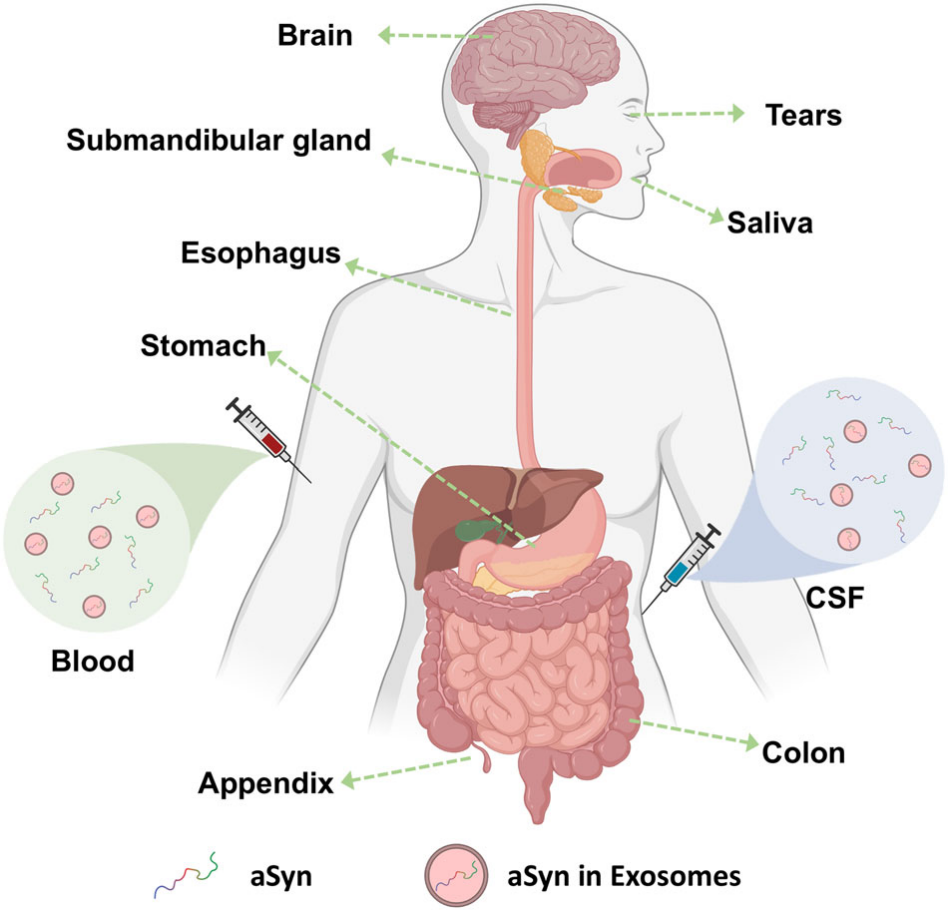
Overview of the localization of aSyn in the human body. Expression of aSyn has been detected in brain tissue, in peripheral tissues as well as in biological fluids. Specifically, in body fluids (blood and CSF), aSyn is circulated both as a free protein and/or exosomes-inbound protein. Created with BioRender.com.

The discovery that aSyn is readily secreted into extracellular spaces and can be found in different forms (monomeric and seeding-competent aggregated forms) in body fluids such as CSF^44–52^, blood components^53–63^, saliva^64–67^ and tears^68,69^ as well as in peripheral tissues (e.g., skin, esophagus, colon)^36,70–72^ sparked even greater excitement about aSyn biomarkers. The diversity of peripheral sources of aSyn presents unique opportunities to develop noninvasive diagnostic and prognostic tools based on measuring the levels of individual or multiple aSyn species (reviewed in refs. ^73–75^) (Fig. 1).

Although various biomarker-based studies (using peripheral tissues and body fluids) have been performed to detect and quantify different aSyn species levels (i.e., total, oligomeric/aggregated or modified forms (those with posttranslational modifications, or PTMs)^73–75^, there is not yet a consensus on which form(s) represent reliable biomarker(s) for early diagnosis, patient stratification or monitoring of disease progression.

Initial studies focusing on quantifying total aSyn levels in CSF reported a general trend of decreased aSyn levels in patients with PD compared to healthy controls (HC)^44–47^. However, several factors have called into question the utility and effectiveness of total aSyn in CSF as a biomarker in clinical practice; these factors include (1) the broad range of aSyn levels reported^44–46,52^, (2) studies reporting no correlation with PD progression/severity as well as a considerable overlap of aSyn levels with controls and other NDDs^45,59,75,76^ and (3) poor interlaboratory reproducibility. Because of these limitations, several groups have pursued modified aSyn species (i.e., aSyn phosphorylated at S129 (pS129)^49,50,53,77–80^ or aggregated forms of aSyn^79,81,82^) as potential biomarkers. Unfortunately, independent replication and validation of many of these studies remain challenging. This has hampered efforts to systematically evaluate and validate the diagnostic value of measuring aSyn species, emphasizing the need for more robust assays that capture the diverse range of aSyn species (total aSyn) or specific modified or aggregated forms of the protein.

The use of specific assays designed to amplify and detect minute amounts of aggregated aSyn in biological samples, termed collectively as aSyn seed amplification assays (aSyn SAAs), (e.g., real-time quaking-induced conversion (RT-QuIC) and protein-misfolding cyclic amplification (PMCA)), have consistently shown the presence of seeding-competent aSyn species in CSF and peripheral tissue biopsies (e.g., from the skin and colon), which could serve as a reliable diagnostic marker for PD with higher accuracy, sensitivity and specificity^35,72,83–87^. These assays do not yet allow reliable discrimination between different synucleinopathies or monitoring of disease progression and severity. However, recent applications of these assays suggest that they could be further optimized and developed to differentiate between PD and other synucleinopathies (MSA from PD and LBD)^36,88^ and potentially identify presymptomatic cases years before they develop PD^34,89^.

### Several aSyn PTMs are closely associated with the progression of pathology in synucleinopathies

PTMs play a key role not only in modulating protein structure and function but also in regulating clearance, localization and secretion. Hence, PTMs could act as molecular switches for regulating biological processes in health and disease. In the context of NDDs such as PD, AD, and amyotrophic lateral sclerosis, PTMs have emerged as key markers of extra- and intracellular inclusions that represent the pathological hallmarks of NDDs. Biochemical studies of LBs, neurofibrillary tangles and other pathological aggregates linked to NDDs have consistently demonstrated that the aggregate-forming proteins (e.g., aSyn, Tau, TDP-43 and amyloid-beta) accumulating in these pathological inclusions or deposits are subjected to a wide range of PTMs (e.g., phosphorylation, glycosylation, acetylation, nitration, SUMOylation, and ubiquitination)^90^. Moreover, many of these PTMs cluster in neighboring sites and compete for the same residue. The close association between specific PTMs (e.g., pS129 aSyn and phosphorylation of Tau at different residues such as Thr181, Ser262 and Ser404) and pathological aggregates^16,91,92^ has led to the emergence of antibodies against such PTMs as the primary tools to detect, monitor and quantify pathology formation in the human brain and animal models of NDDs. However, our understanding of the precise role of PTMs in regulating protein misfolding, aggregation, and the development and spread of pathology in NDDs remains incomplete. Interestingly, recent findings suggest that most of PTMs seen in pathological aggregates may occur after aSyn aggregation and could be involved in regulating the processing of fibrils and/or the formation and maturation of LBs (see recent reviews^93–95^). This suggests that they could play important roles in regulating the secretion of aSyn aggregates and influence their seeding activity in the CSF. Therefore, their detection in biological fluids may indeed provide a window to pathological aSyn in the brain (Boxes 1 and 2).

Several aSyn PTMs have been identified in the postmortem brain tissues of patients with PD and other synucleinopathies using different approaches such as mass spectrometry (MS) and antibody-based assays (e.g., immunohistochemistry)^16,17,96–99^. Among the most frequently reported aSyn PTMs are acetylation (at the N-terminus and lysine residues), ubiquitination, phosphorylation (at S129 and, to a lesser extent, at Y39, S87, Y125), and nitration (at Y39, Y125, Y133, Y136), as well as several N-terminal and C-terminal truncations^16,17,96–99^. In addition, pS129 aSyn species have been detected in several peripheral tissues, including the skin, esophagus and colon, of patients with PD and synucleinopathies^100–103^, although the correlation between pS129 levels and peripheral aSyn pathology remains unclear, mainly because most studies describe the detection of pS129 immunoreactivity without assessing the aggregation state of aSyn.

**Fig. 2:**
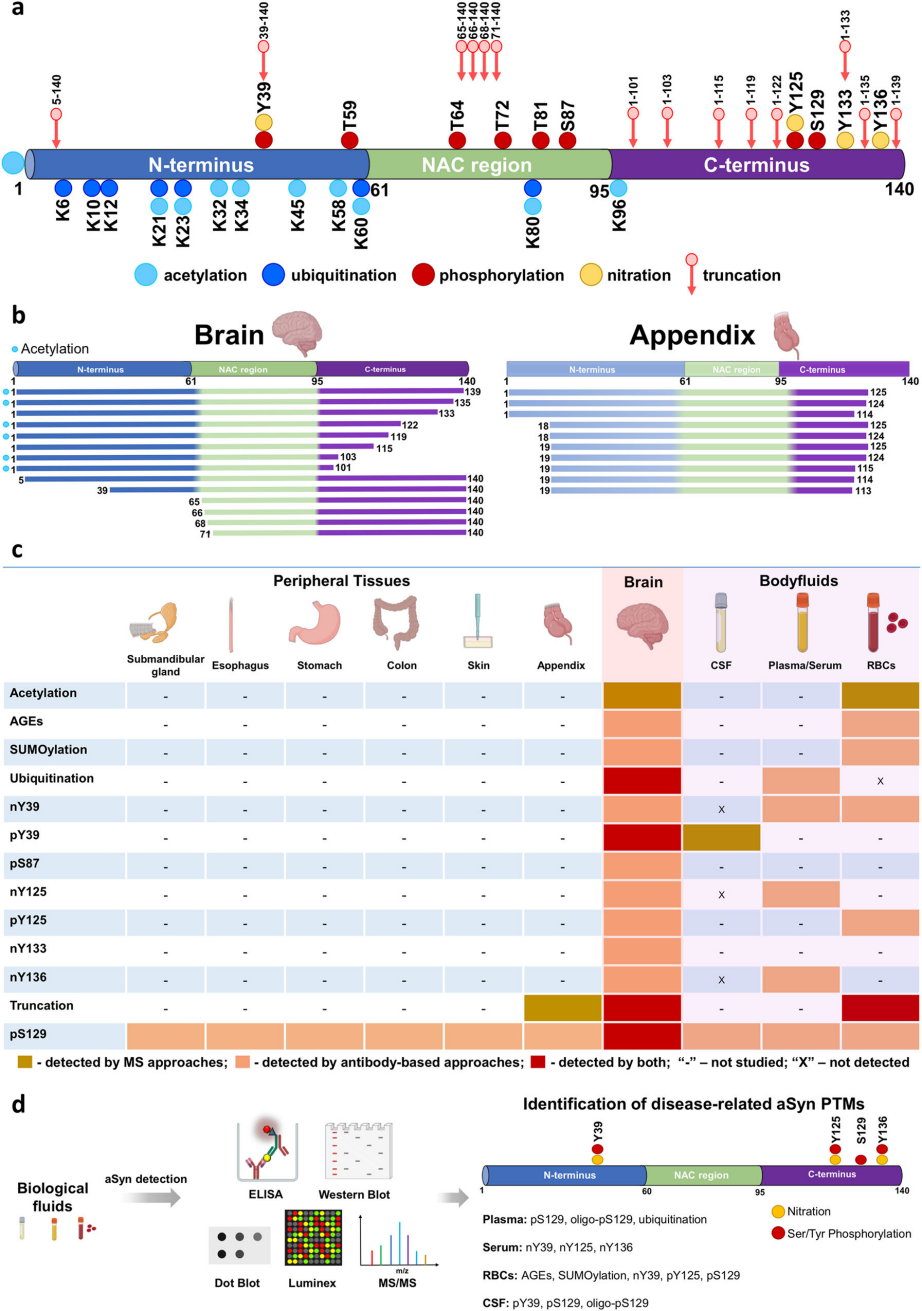
Schematic illustration of aSyn PTMs identified in biological specimens from human synucleinopathy patients. **a** A schematic depiction of aSyn PTMs detected in the brains of patients with synucleinopathies by MS studies and immunohistochemistry. **b** Comparison of the truncated aSyn species identified in the brain and appendix identified by MS and antibody-based approaches. **c** Overview of aSyn PTMs identified in the peripheral tissues, brain and body fluids. Various aSyn PTMs have been reported to be present in the human brain using different approaches, i.e., MS or antibody-based assays. Remarkably, some of these PTMs have also been identified in different peripheral tissues and body fluids. pS129 aSyn is the most extensively studied disease-related PTM and is reported to be present in various biological specimens. **d** PTMs of human aSyn in body fluids and assays used to profile, identify and quantify unmodified and modified aSyn species. Schematic depiction of the aSyn PTMs identified in different constituents of the blood [plasma, serum and red blood cells (RBCs)] and CSF.

Among the many aSyn PTMs found in the brain, pS129, truncations, and ubiquitination are the most commonly detected PTMs and correlate with pathology formation^90^ (Fig. 2a). However, most studies have focused mainly on exploring the role of primarily pS129 aSyn in the pathogenesis of synucleinopathies and its potential as a PD-related biomarker. This is primarily because it is one of the most abundant PTMs but also because several antibodies against this PTM are available, whereas only few antibodies are available for other modified aSyn species, such as ubiquitinated and truncated forms.

Increasing evidence point to N- and C-terminal truncations as the second most common type of aSyn PTMs in the brains of patients with PD and other synucleinopathies^16,104–106^, truncated aSyn species have also been detected in the soluble fraction of homogenates from healthy human brains, suggesting that they may be involved in regulating some physiological functions of aSyn^16,96–99^. C-terminal truncations of aSyn are highly enriched in the pathological inclusions of different synucleinopathies, such as PD^16,99,104,105,107–116^, DLB^16,99,106–108,111,114,117^, and MSA^16,104,113,118^, and in AD patient brains without LB pathology^110^ compared to the insoluble fraction from control subjects. Although the majority of these studies relied primarily on antibodies and Western blotting analyses, the presence of C-terminal truncations has been further verified by unbiased tandem mass spectrometry (MS/MS) approaches. These studies revealed the presence of C-terminal truncations in PD^96,97,99^, DLB^16^, MSA^17^ and PD with dementia^99^ (Fig. 2a). Among the most representative truncated species were aSyn 1–119 and 1–122^16,17,97,99^. Recently, N-terminal truncations (5–140, 39–140, 65–140, 66–140, 68–140 and 71–140) (Fig. 2a, b) have also been detected in human LBD brains^106^ and PD brains^96,97^; however, the pathological relevance of these N-terminally truncated forms of aSyn remains unclear. Surprisingly, aSyn truncations have also been reported to be present in the human appendix^109^, with the great majority of truncated aSyn species being cleaved in both the N- and C-terminal regions of the protein. However, a comparison of the various aSyn species detected in the brain and the appendix (Fig. 2b) reveals major differences in the cleavage sites and distribution of truncated aSyn species^16,96,97,99,109^. Figure 2c summarizes the different PTMs identified in the brain, biological fluids and peripheral tissues.

The close association of different modified and aggregated forms of aSyn with pathological aSyn inclusions in the brain of patients with PD and other synucleinopathies has led to increasing interest in potentially quantifying the levels of these species in biological fluids as potential disease biomarkers. This section presents a comparative and critical analysis of previous studies to measure total aSyn and posttranslationally modified, and aggregated (oligomer and fibril) forms of aSyn in different biological fluids.

Box 1 Summary of the main findings of the studies that aimed to evaluate the potential value of aSyn as a biomarker for PD and other synucleinopathies

- Although total aSyn levels in CSF consistently show a trend of reduction in PD patients compared to controls, the reported levels vary significantly from one study to another.
- aSyn PTMs are relatively scarce in biological fluids but are abundant in pathological aggregates in the brain and peripheral tissues and may constitute the predominant form of aSyn in specific cells (e.g., astrocytes).
- The great majority of studies have focused primarily on the detection and quantification of pS129, which is readily detected in appreciable amounts in the blood components (plasma or red blood cells) but not in the CSF.
- The detection of various modified forms of aSyn increases in biological fluids containing higher concentrations of aSyn (e.g., red blood cells compared to other blood components or CSF).
- Most studies on aSyn levels or PTMs have relied on the use of selected antibodies targeting specific species rather than an unbiased approach aimed at profiling all aSyn species, and in most cases, the antibodies have not been validated for their ability to capture the diversity of modified aSyn species.
- Oligomeric aSyn levels are reported to be increased in PD patients across the different biological fluids. However, the sensitivity, specificity, and reproducibility of assays for oligomeric aSyn forms remain unsatisfactory.
- The great majority of the antibodies and assays used to quantify aSyn oligomers do not allow differentiation among oligomeric, fibrillar and other aggregated forms of aSyn. Therefore, it remains unclear what form or forms of the protein are measured in these studies.
- Only two studies have investigated the presence of levels of posttranslationally modified oligomeric forms of aSyn in biological fluids.
- The lack of standardization in aSyn protein concentration determination methods and the reliance on poorly characterized protein standards are major contributors to the large variations in the quantification of total and modified aSyn levels measured in different studies.

Box 2 Main recommendations for aSyn-biomarker studies

1. Develop optimized protocols for sample collection and handling that take into account the stability of the different modified forms of aSyn.
2. Use multicenter cohorts and a large number of biological fluid samples from PD and other synucleinopathies.
3. Use highly pure and homogeneously modified protein standards.
4. Use amino acid analysis as the gold standard method for determination of aSyn protein concentration.
5. Use well-characterized and validated antibodies that detect the specific forms of the protein (PTM specific) or capture the diversity of aSyn species (for total aSyn concentration determination).
6. Generate new antibodies against different aSyn PTMs, as the current antibody toolsets cover only a limited number of aSyn PTMs.
7. Assess the cross-reactivity of the available and newly generated antibodies (particularly pS129) to other aSyn PTMs and the proteome of biological fluids.
8. Use a combination of biochemical and mass spectrometry approaches, to conduct systematic and unbiased analysis of aSyn species and PTMs in biological fluids, peripheral tissues and postmortem brain tissues from healthy controls and patients with PD and other synucleinopathies and NDDs (e.g., PDD, MSA, DLB, AD).
9. Develop a diagnostic workflow that integrates biochemical and structural aSyn biomarkers and other biomarkers of disease-relevant mechanisms (neurodegeneration, synaptic dysfunction, inflammation, etc.).

### aSyn PTMs in biological fluids and their potential as biomarkers

The close association between aSyn pathology in the brain and several aSyn PTMs, combined with converging findings demonstrating that neurons secrete different forms of aSyn, has led to the pursuit of modified aSyn species in different peripheral tissues (e.g., appendix, skin, colon and esophagus)^100–103,109^ and biological fluids^73,75^ (Fig. 2c) as potential biomarkers of peripheral pathology or PD. The assumption here is that the distribution of aSyn species in biological fluids, such as CSF or blood, may provide a window to the dynamics of aSyn proteoforms in the PD brain and reveal changes that reflect the extent of pathology and/or disease progression. Over the past decades, various methods and assay platforms have been employed to profile, detect and quantify modified aSyn isoforms in body fluids (Fig. 2d), including Western blot (WB); dot blot; Enzyme-linked immunosorbent assay (ELISA); biotin ELISA; phospholipid ELISA; electrochemiluminescence (ECL); immunomagnetic reduction-based, Luminex, and Singulex assays; MS; and modified paired surface plasma wave biosensors (PSPWB) coupled to immunoassays.

In this review, we will (1) provide an overview of the progress made toward mapping aSyn PTMs in different biological fluids (CSF, plasma, serum, and RBCs); (2) present critical analyses of previous studies that have sought to explore the potential of total aSyn and aSyn PTMs as biomarkers to monitor disease progression or differentiate between PD patients and HC or between PD and other synucleinopathies; (3) present a gap analysis to help guide future aSyn biomarker studies; (4) highlight some of the current challenges in targeting modified and aggregated aSyn species as potential biomarkers for PD; and (5) provide an overview of how recent advances in protein synthesis and more sensitive approaches (MS) to detect PTMs may help address these challenges. Finally, we outline a series of specific recommendations for the design of future biomarker studies, sample handling, and research tool development and validation that we believe will pave the way to develop sensitive and accurate assays that capture and more accurately measure the diversity of aSyn forms in biological fluids and samples. The need for a more integrative approach that combines multiple biomarkers linked to different disease mechanisms implicated in PD and other synucleinopathies is also discussed. Table 1 summarizes the different studies on aSyn PTM biomarkers of PD and other synucleinopathies using biological fluids; these studies will be described in detail in the next sections.

**Table 1:** Analysis of aSyn PTMs species in the different biological fluid specimens: overview of the different techniques, antibodies employed and aSyn PTMs concentration range across control and patient groups.

PTM	Biological fluid	Techniques employed	Antibody or enrichment strategy used (Vendor)	Mean of modified aSyn in controls	Mean of modified aSyn in cases	Preparation of calibrants	Methods used for calibrant characterization	Calibrant purity and characterization—Data shown	References
nY39	Red blood cells	Immunoblotting analysis (Western blot and dot blot)	Anti-nitro-α/β-Synuclein, nY39, 36-012 (Upstate/Millipore)	NA	NA	NA	NA	NA	Vicente Miranda et al.^146^
Nitrotyrosine	Serum	ELISA, Western Blot and Mass spectrometry	Monoclonal antibody to nitrotyrosine (Hycult Biotech); Nitrosylated aSyn (nY125/136): anti-nitro-a/b-synuclein antibody - nSYn12 (Millipore); Nitrosylated aSyn (nY39): anti-nitro-a/b-synuclein antibody Tyr39 - nSYn14 (Millipore)	3-nitrotyrosine protein: 48.3 ± 6.8 nM	70.6 ± 4.7 nM (Serum)	NA	NA	NA	Fernandez et al.^145^
pY39	CSF	Targeted Mass spectrometry	PTMScan Phospho-Tyrosine antibody; P-Tyr-1000 (Cell Signaling Technology); TiO_2_ beads (Thermo Fisher)	1.67–4.98 attomole/ml	PD: 1.53–4.25 attomole/ml	Synthetic	Synthesis	NA	Na et al.^142^
pY125	Red blood cells	Immunoblotting analysis (Western blot and dot plot)	ab10789 (Abcam)	NA	NA	NA	NA	NA	Vicente Miranda et al.^146^
pS129	CSF	IP-MS, Luminex	IP-MS: pS129 (Abcam) and ExactaCruz IP kit; Luminex: Capture: ASY-1^a^; Detection: biotinylated anti-human pS129^a^	68.61 ± 17.25 to 73.03 ± 17.20 pg/ml	PD: 77.73 ± 20.45 to 79.23 ± 23.22 pg/ml	Recombinant aSyn was incubated with casein kinase II (New England Biolabs)	Immunoblotting with a phosphorylation-dependent anti-aSyn antibody, pS129 (Epitomics) and mass spectrometry	MALDI-TOF/MS	Wang et al.^50^
					MSA:58.12 ± 20.24 to 61.97 ± 14.19 pg/ml				
					PSP: 55.54 ± 16.87 to 58.24 ± 24.93 pg/ml				
					AD: 67.50 ± 15.68 to 72.64 ± 19.57				
	CSF	ELISA	Capture: anti-α-synuclein N-19 (Santa Cruz Biotechnology); Detection: anti-pS129 (Epitomics)	3.58 ± 3.85 μg/ml	PD: 3.43 ± 6.18 μg/ml	Recombinant aSyn was incubated with casein kinase II (New England Biolabs)	Immunoblotting with a phosphorylation-dependent anti-aSyn antibody, pS129 (Epitomics) and mass spectrometry	NA	Foulds et al.^49^
					PD (nonD): 4.41 ± 8.68 μg/ml				
					PD (Cog): 1.76 ± 1.02 μg/ml				
					PD (Dem): 3.67 ± 5.73 μg/ml				
					DLB: 1.63 ± 1.42 μg/ml				
					PSP: 5.14 ± 9.73 μg/ml				
					MSA: 7.14 ± 9.19 μg/ml				
	CSF	Luminex	Biotinylated anti-human pS129 antibody^a^ Streptavidin-R-PE (Prozyme)	NA	Baseline: 114.66 ± 17.14 (pg/ml)	Recombinant aSyn was incubated with casein kinase II (New England Biolabs)	Immunoblotting with a phosphorylation-dependent anti-aSyn antibody, pS129 (Epitomics) and mass spectrometry	NA	Stewart et al.^77^
					Follow-up: 117.89 ± 17.92 (pg/ml) PD (UW-collaborative): 74.01 ± 26.67 (pg/ml) LRRK2: 63.79 ± 22.73 (pg/ml)				
	CSF	ELISA, dot plot	Mouse anti-pS129-α-syn monoclonal antibody^a^	222 (180.5–275) pg/ml	261 (206.8–296.3) pg/ml	Methodology is not described	Not described in the method section	NA	Majbour et al.^79^
	CSF	ELISA	Mouse anti-pS129-α-syn monoclonal antibody^a^	NA	Baseline: 220.2 (145.0–316.4) pg/ml	Methodology is not described	Not described in the method section	NA	Majbour et al.^78^
					Follow-up: 180.8 (125.0–252.2) pg/ml				
	CSF	ELISA	Mouse anti-pS129-α-syn monoclonal antibody^a^	116 (103–145) pg/ml	Sporadic PD: 139 (114.25–163) pg/ml	Methodology is not described	Not described in the method section	NA	Majbour et al.^80^
					Asymptomatic LRRK2 mutation carriers: 121 (94–150) pg/ml				
					Symptomatic LRRK2 mutation carriers: 122 (106–145) pg/ml				
					Follow-up: 117.89 ± 17.92 (pg/ml)				
	CSF	ELISA	Mouse anti-pS129-α-syn monoclonal antibody^a^	235 ± 54 pg/ml	PD: 258 ± 52 pg/ml	Methodology is not described	Not described in the method section	NA	van Steenoven et al.^128^
					DLB: 232 ± 79 pg/ml				
					AD: 220 ± 61 pg/ml				
	CSF	ELISA	Mouse anti-pS129-α-syn monoclonal antibody^a^	NA	PD: 85 (55–110) pg/ml	Methodology is not described	Not described in the method section	NA	Constantinides et al.^129^
					MSA: 54 (46–64) pg/ml				
					PSP: 67 (56–78) pg/ml				
					CBD: 60 (53–109) pg/ml				
					AD: 59 (47–79) pg/ml				
					FTD: 49 (34–72) pg/ml				
					VD: 55 (46–93) pg/ml				
	CSF	ELISA using the Erenna Immunoassay System	Capture: PRTA-11A5 Detection: PRTA-23E8	2.19 ± 0.83 pg/ml	PD: 1.94 ± 0.90 pg/ml	Recombinant aSyn was incubated with PLK2	Mass spectrometry	NA	Schulz et al.^131^
					MSA: 1.84 ± 0.71 pg/ml				
					DLB: 2.34 ± 0.97 pg/ml				
					FTD/ALS: 2.28 ± 1.04 pg/ml				
					AD: 2.45 ± 1.08 pg/ml				
					CBS: 2.07 ± 0.83 pg/ml				
					PSP: 1.95 ± 0.90 pg/ml				
	CSF	ELISA	Mouse anti-pS129-α-syn monoclonal antibody^a^	Baseline: 112 (89–129) pg/ml 24 months follow-up: 101 (75–131) pg/ml 48 months follow-up: 98 (89–140) pg/ml	Baseline: 116 (89–160) pg/ml 24 months follow-up: 105 (78–126) pg/ml 48 months follow-up: 128 (92–174) pg/ml	Methodology is not described	Not described in the method section	NA	Majbour et al.^132^
	CSF	ELISA	Mouse anti-pS129-α-syn monoclonal antibody^a^	225 (185–279) pg/ml	265 (208–296) pg/ml	Methodology is not described	Not described in the method section	NA	Oosterveld et al.^130^
	Plasma and CSF	IP, Western Blot and Singulex Assays	MJF-R13 (8-8) (ab168381; Abcam)	Plasma: 878.5 ± 317.4 pg/ml	NA	Recombinant aSyn was incubated with PLK3	UPLC, mass spectrometry, SDS-PAGE and WB analysis using pS129 antibody (ab168381)	NA	Cariulo et al.^53^
				CSF: NA (below detection limit)					
	Plasma	Western blot and biotin ELISA	ELISA- Capture: anti-α-synuclein N-19 (Santa Cruz Biotechnology); Detection: anti-pS129 (Epitomics); Western blot: pS129 (Epitomics)	0.15–0.6 ug/ml	PD:0.2–2 ug/ml	Recombinant aSyn was incubated with casein kinase II (New England Biolabs)	Immunoblotting with a phosphorylation-dependent anti-aSyn antibody, pS129 (Epitomics) and mass spectrometry	NA	Foulds et al.^54^
	Plasma	ELISA	Capture: anti-α-synuclein N-19 (Santa Cruz Biotechnology); Detection: anti-pS129 (Epitomics)	143.4 ± 531.8 ng/ml	756.8 ± 2419.9 ng/ml	Recombinant aSyn was incubated with casein kinase II (New England Biolabs)	Immunoblotting with a phosphorylation-dependent anti-aSyn antibody, pS129 (Epitomics) and mass spectrometry	NA	Foulds et al.^55^
	Plasma	Immunomagnetic reduction (IMR)-based immunoassay	Dextran-coating magnetic Fe3O4 nanoparticles (MF-DEX-0060, MagQu) bio-functionalized with monoclonal antibody - 825701, (Biolegend)	0.8 ± 0.6 fg/ml	12.9 ± 8.7 fg/ml	Synthetic: pS129 aSyn peptide (ab188826)	Synthesis^b^	NA	Lin et al.^143^
	Serum	Modified paired surface plasma wave biosensor coupled to an immunoassay and non-labeled technique	Rabbit monoclonal anti-α-syn (phosphor S129) antibody (anti-p-S129-α-syn; Abcam, Cambridge, MA, USA)	0.5–5 ng/ml	4–12 ng/ml	Human phosphorylated aSyn ELISA kit (MyBioSource Co., Vancouver, Canada)	NA	NA	Chen et al.^144^
	Red blood cells	Phospholipid-ELISA assay	pSyn#64 (WAKO)	24.48 ± 7.6 pg a-Syn/mg protein	PD-M: 35.820 ± 15.19 pg a-Syn/mg protein	Human pS129 aSyn (RP-004; Proteos)	Semisynthetic^b^	NA	Elhadi et al.^150^
					PD-D: 27.370 ± 9.76 pg a-Syn/mg protein				
	Red blood cells	ELISA, Western blot and immunodepletion	Nonbio sc-135638 (Santa Cruz Biotechnology)	11.89 ± 3.57 ng/mg	MSA: 14.02 ± 4.02 ng/mg MSA-P:13.27 ± 1.91 ng/mg MSA-C: 12.19 ± 3.04 ng/mg	Recombinant aSyn was incubated with casein kinase II (New England Biolabs)	SDS-PAGE and Western blotting analysis with a phosphorylation-dependent anti-aSyn antibody	NA	Li et al.^151^
	Red blood cells	Electrochemiluminescence assay	Anti-pS129 (BioLegend)	Cytosol: 67.36 ± 0.48 pg/μg	Cytosol: 636.05 ± 6.03 pg/μg	Human Alpha-synuclein pS129 (RP-004; Proteos)	Semisynthetic^b^	NA	Tian et al.^149^
				Membranes: 255.05 ± 1.98 pg/μg	Membranes: 315.35 ± 0.95 pg/μg				
Oligo-phosphorylation	CSF	ELISA	Capture: anti-pS129 (Epitomics); Detection: biotinylated pS129^a^	1.05 ± 2.23 μg/ml	PD: 0.77 ± 1.51 μg/ml	Recombinant aSyn was incubated with casein kinase II (New England Biolabs)	Immunoblotting with a phosphorylation-dependent anti-aSyn antibody, pS129 (Epitomics) and mass spectrometry	NA	Foulds et al.^49^
					PD (nonD): 0.26 ± 0.03 μg/ml				
					PD (Cog): 0.68 ± 0.78 μg/ml				
					PD (Dem): 1.28 ± 2.27 μg/ml				
					DLB: 1.60 ± 3.02 μg/ml				
					PSP: 1.25 ± 3.32 μg/ml				
					MSA: 19.56 ± 1.66 μg/ml				
	Plasma	Biotin ELISA	Capture: anti-pS129 (Epitomics); Detection: biotinylated pS129^a^	HC: 0.04–0.09 ug/ml	PD: 0.04–0.18 ug/ml	Recombinant aSyn was incubated with casein kinase II (New England Biolabs)	Immunoblotting with a phosphorylation-dependent anti-aSyn antibody, pS129 (Epitomics) and mass spectrometry	NA	Foulds et al.^54^
Advanced glycation end-products (AGEs)	Red blood cells	Immunoblotting analysis (Western blot and dot blots)	AGEs KAL-KH001 (Cosmo-Bio)	NA	NA	NA	NA	NA	Vicente Miranda et al.^146^
SUMOylation	Red blood cells	Immunoblotting analysis (Western blot and dot blot)	Sc-9060 (Santa Cruz Biotechnology)	NA	NA	NA	NA	NA	Vicente Miranda et al.^146^
Ubiquitination	Plasma	Western blot and biotin ELISA	Anti-ubiquitin antibody FL-76 (Santa Cruz Biotechnology)	NA	NA	NA	NA	NA	Foulds et al.^54^
	Red blood cells	Immunoblotting analysis (Western blot and dot blot)	ab24686 (Abcam)	NA	NA	NA	NA	NA	Vicente Miranda et al.^146^

*NA* not applicable.

^a^Antibodies generated in-house.

^b^Commercial available.

### aSyn in the CSF

CSF remains one of the main accessible body fluids and provides a window to biochemical and neuropathological changes in the brain. Therefore, it is not surprising that the search for aSyn biomarkers has focused primarily on CSF, especially in the absence of validated aSyn-pathology-specific brain positron emission tomography tracers and biomarkers. Initial studies focused on measuring total aSyn levels, but interest in aggregated and phosphorylated forms (namely, pS129) increased over time, with converging evidence suggesting links between these species and aSyn pathology in the brains of patients with PD and other synucleinopathies.

#### Total aSyn levels in the CSF

**Fig. 3:**
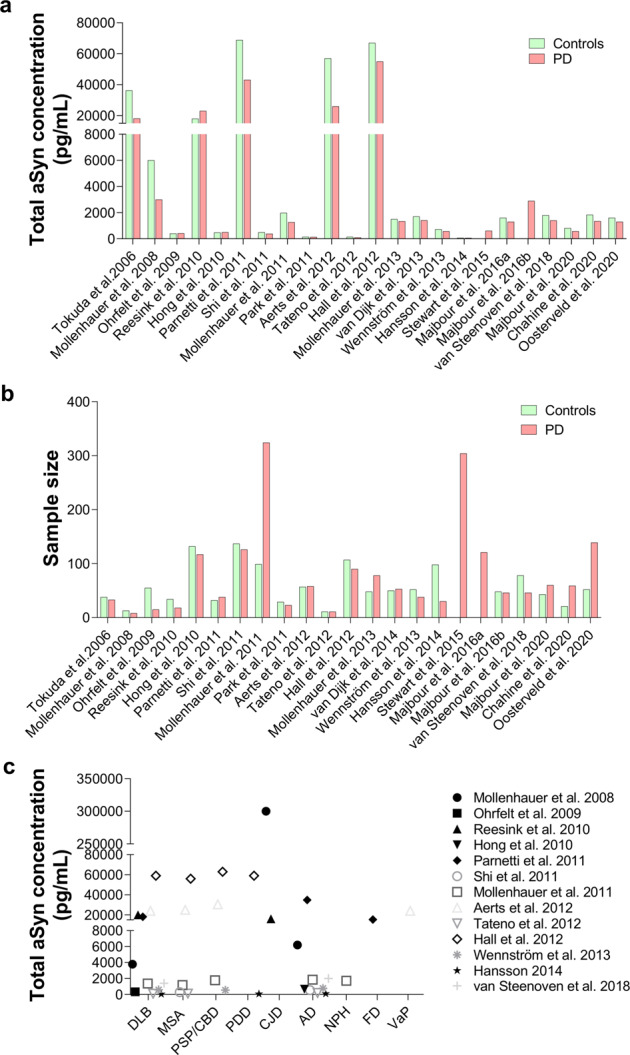
An overview of previous studies reporting total aSyn levels in CSF of PD, controls and patients suffering from other synucleinopathies (e.g., DLB, MSA, PSP) and tauopathies. **a** Comparison of CSF levels of total aSyn between PD and controls in different studies. **b** Sample size of PD and controls used in the studies shown in a. **c** Variability of CSF levels of total aSyn across other disease-group patients. Each dot in the graph displays the average values of aSyn reported in the respective study. CBD corticobasal degeneration, MCI mild cognitive impairment, VaP vascular parkinsonism, CJD Creutzfeldt–Jakob disease, NPH normal-pressure hydrocephalus, FD frontotemporal dementia.

Although aSyn is consistently detected in the CSF, mainly using ELISA and other immunoassays, the reported levels of CSF aSyn in control individuals and patients suffering from PD or other synucleinopathies vary significantly from one study to another^44–52^. For example, levels of total aSyn were reported to be in the range of 67–68,900 pg/ml and 61.5–55,000 pg/ml in controls and PD patients, respectively^44–47,52,119–121^ (Fig. 3a and Table 2). The majority of these studies had small sample sizes, and, remarkably, the numbers of samples derived from PD patients and controls used did not increase across the years (Fig. 3b). In other synucleinopathies, the reported total aSyn levels are as follows: (1) DLB: 58–59,000 pg/ml; 1420 ± 1260 pg/ml, (2) MSA: 108–56,000 pg/ml and (3) progressive supranuclear palsy (PSP): 428–63,000 pg/ml^44,45,50,119–121^ (Fig. 3c). A single study by Foulds et al. reported much higher levels of total aSyn, specifically in the range of µg/ml instead of the observed pg/ml, for patients suffering from different synucleinopathies as well as control subjects (PD: 1.85 ± 2.40 µg/ml (average); DLB: 2.31 ± 2.51 µg/ml; MSA: 3.80 ± 2.40 µg/ml; PSP: 1.45 ± 1.97 µg/ml and HC: 1.87 ± 2.29 µg/ml)^49^. These large variations have been attributed to several preanalytical and analytical confounding factors as well as to clinical and demographic data heterogeneity, comorbidities and potential medical treatments that have been extensively discussed in recent reviews (see refs. ^75,76,122^). This has led to inconclusive findings regarding differences in aSyn levels between PD patients and other parkinsonism patients and whether changes in total aSyn levels could be used to monitor disease progression. For example, several cross-sectional studies showed a decrease in total aSyn levels in PD patients^44–47^ compared to HC individuals and controls with other NDDs, which was corroborated to some extent by a number of meta-analysis studies^51,123–125^. Nonetheless, several studies did not show that there was any correlation between total aSyn levels and disease progression/severity or that changes in aSyn levels provided a reliable marker that distinguishes between PD and other synucleinopathies, such as MSA or PSP^45,59,76^. However, two studies comparing PD patients with controls reported an association with PD severity/progression^78,126^. Furthermore, in a recent meta-analysis study, Eusebi et al. reported that aSyn levels did not differentiate PD from other types of parkinsonism^51^. One consistent finding in the majority of these studies is that aSyn can be reliably detected in the CSF (Fig. 3a).

**Table 2:** Overview of the concentration of unmodified aSyn and pS129 aSyn in the different biological fluid specimens.

Biological fluid	aSyn levels range in controls and PD	pS129 levels range in controls and PD	Other PTMs detected
CSF	61.05–68,900 pg/ml^44–47,52,119–121^; 1.34 ± 2.16–3.80 ± 2.40 µg/ml^49^	No detection to 7.14 ± 9.19 µg/ml^49–51,53,77,79,80,128–130^	pY39; oligo-pS129
Plasma	3600–1,777,100 ± 3,609,600 pg/ml^53–59^	0.8 ± 0.6 fg/ml–12.9 ± 8.7 fg/ml^143^; 878.5 ± 317.4–756,800 ± 2,419,900 pg/ml^53,55^	oligo-pS129; ubiquitination
Red blood cells	26,200 ± 3000–40,000 ng/ml^60–62^	24.48 ± 7.6–636,050 ± 6030 pg/mg^149–151^	AGEs; SUMOylation; nY39; pY125
Saliva	7.104 ± 5.122–314.03 ± 435.9 pg/ml^64–66^; 159.4 ± 61.6–229.9 ± 64 ng/ml^67^	NA	NA
Tears	32.02–361.16 pg/mg^68,69^	NA	NA

*NA* not applicable.

In addition to the confounding factors highlighted above, differences in the distribution of modified aSyn forms could also contribute to the large variations in aSyn levels, especially since the great majority of the capture and/or detection antibodies used in most aSyn immunoassays target the C-terminal domain of aSyn (residues 110–130), which harbors the most abundant pathology-associated aSyn modifications (e.g., phosphorylation and C-terminal truncations; Fig. 2a).

#### pS129

**Fig. 4:**
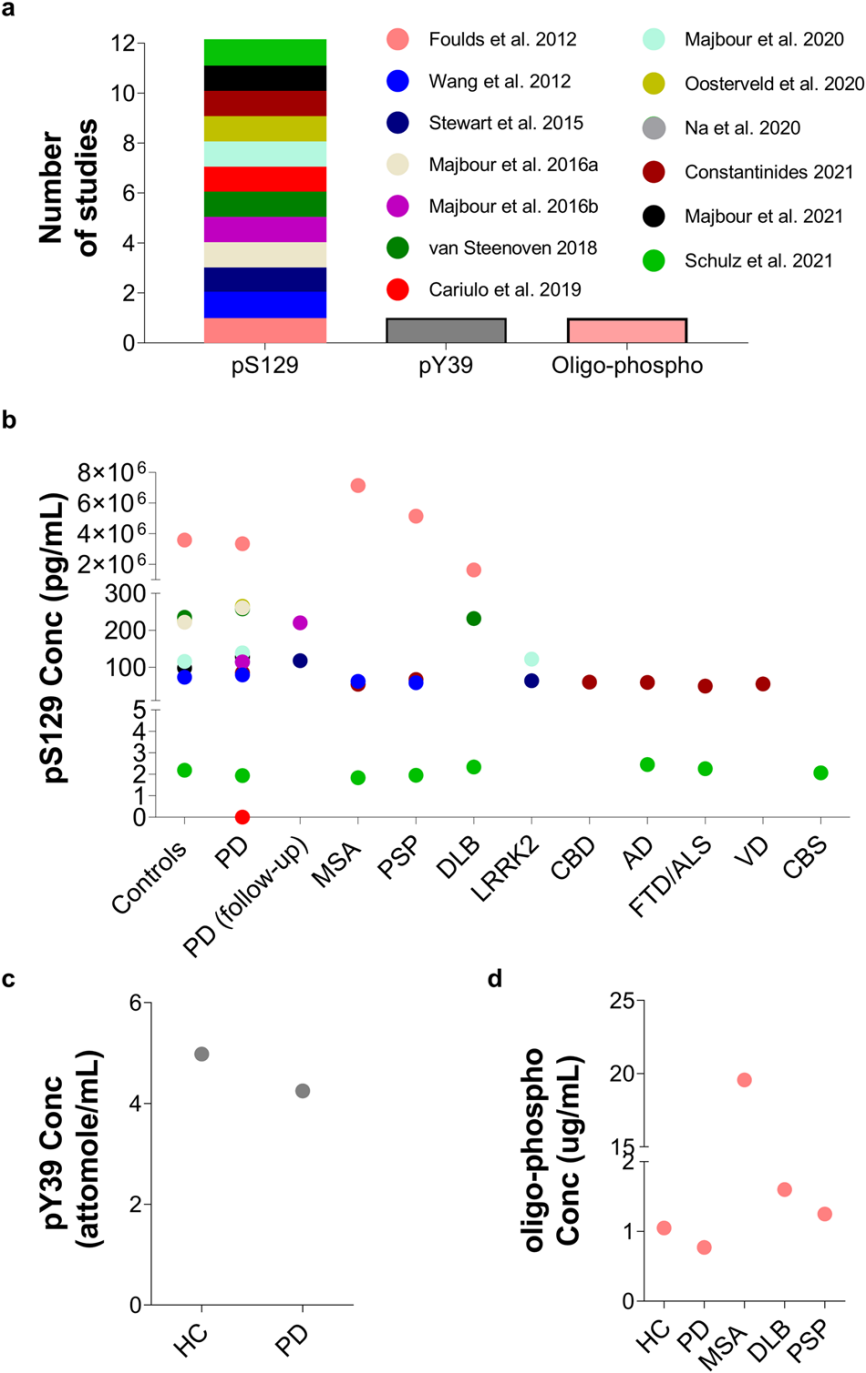
aSyn PTMs in CSF. **a** Number of studies that identified CSF aSyn PTMs. **b** Variability of pS129 levels across the different disease groups and controls. Each dot in the graph corresponds to the average values of pS129 aSyn reported in the respective study. Other aSyn PTMs, i.e., **c** Levels of pY39 and **d** oligo-phosphorylation reported in single studies. The legend/color code in **a** corresponds also to **b**, **c** and **d**.

The search for posttranslationally modified forms of aSyn in CSF initially focused on pS129 aSyn because of the converging evidence demonstrating that pS129 is the predominant modified form of aSyn in LBs (reviewed in ref. ^127^) and a reliable marker of pathology that correlates with the increase in pathology formation in the brain^49,50,53,77–80^. Several studies suggested that CSF pS129 levels might enable the diagnosis of PD^50,51,77,79^ or differentiate not only between PD patients and control subjects but also between different synucleinopathies^49,128,129^ (Fig. 4a). Nevertheless, the results from these studies revealed high variability in terms of the detected pS129 levels in the CSF, ranging from no detection to 7.14 ± 9.19 µg/ml^49,53^ (Fig. 4b).

When comparing the CSF pS129 levels between HC and PD patients, several studies reported a significant elevation in the latter^50,51,77,79,130^. One study suggested that the observed change in pS129 levels allows differentiation between diagnostic cohorts^50^. However, other studies observed no significant change in the mean pS129 values between HC and PD patients^49,80,128,131^ or between patients with PD and those with other synucleinopathies^49,128,129^ (such as DLB^49,128^, MSA or PSP^49,129^) or tauopathies^128,129^. One study reported the following pS129 concentrations in different subgroups: MSA (7.14 ± 9.19 µg/ml); PSP (5.14 ± 9.73 µg/ml); DLB (1.63 ± 1.42) and controls (3.58 ± 3.85 µg/ml). Despite the lack of significant differences, MSA and PSP patients showed increased CSF pS129 values, while DLB patients showed reduced levels in comparison to healthy individuals^49^. Furthermore, pS129 levels were slightly increased in MSA patients than in PSP patients and even higher than in DLB patients^49^. However, in another report, Wang and collaborators evaluated pS129 levels in samples from PD, MSA and PSP patients along with controls, and reported values on the scale of pg/ml^50^. This study revealed reduced pS129 levels in patients suffering from MSA and PSP in comparison with PD patients and controls. Interestingly, they observed similar concentration levels between the MSA and PSP disease groups, while the PSP cohort displayed significantly lower pS129 levels than controls^50^.

The correlation between pS129 aSyn levels and disease severity was also assessed in several studies, which yielded inconsistent results. Wang et al.^50^ reported a correlation of decreased pS129 levels with PD severity, which was further corroborated by others^77,78^, suggesting that pS129 in the CSF may serve as a progression biomarker for PD and is associated with different PD clinical phenotypes. However, a study by Majbour et al.^79^ did not show any association between pS129 levels and disease severity and progression. Moreover, in a recent study, Majbour et al. reported that the pS129 aSyn levels of PD patients were lower at the 2-year follow-up than at baseline; however, this observed reduction did not display statistical significance^132^.

It has been suggested that the ratios of pS129 to total aSyn and/or oligomeric aSyn to total aSyn, as opposed to pS129 levels only, may provide a superior diagnostic marker and could differentiate between PD and other synucleinopathies^50,77,80,81,133,134^. Two studies reported an elevated ratio of pS129 aSyn to total aSyn in PD and symptomatic and asymptomatic LRRK2 mutation carrier patients compared with controls^50,80^. The level of pS129 aSyn/total aSyn was also shown to increase with disease progression in PD patients in two different follow-up studies^77,78^, enabling the differentiation between MSA and PSP patients^50^. To the best of our knowledge, only one study has sought to assess the level of phosphorylated aggregated forms of aSyn in the CSF. Remarkably, in this study, the authors reported that the phosphorylated oligomeric form of aSyn varied among the PD, DLB, PSP, MSA and control groups, with highly significant differences reported in MSA (19.56 ± 1.66 μg/ml) in comparison with all the other groups (PD: 0.75 ± 1.15 μg/ml (average); DLB: 1.60 ± 3.02; PSP: 1.25 ± 3.32 μg/ml; and controls: 1.05 ± 2.23 μg/ml) (Table 1)^49^. Interestingly, the level of the phosphorylated oligomeric form of aSyn was marginally decreased in PD patients in comparison to controls (Fig. 4a, c) as well as DLB and PSP patients. Furthermore, DLB and PSP patients displayed slightly higher levels than controls, while the DLB group displayed marginally higher levels than PSP patients. However, in MSA patients, this aSyn species was estimated to be at an ~20-fold higher concentration than in other diseased patients (Fig. 4c)^49^. Surprisingly, the levels of pS129 detected in CSF have displayed great variability^49,50,77–80,128–130^ (see Table 1).

Recently, Cariulo et al.^53^ developed the Singulex Erenna immunoassay, an ultrasensitive immunoassay based on a quantitative fluorescent sandwich immunoassay coupled to single-molecule counting technology, for quantifying total aSyn and pS129 species in the range of pg/mL. They reported that pS129 could be readily detected in human plasma (at ~878.5 ± 317.4 pg/ml) but not in CSF. This sensitive assay could detect recombinant and homogeneous pS129 aSyn with a detection limit of 0.15 pg/ml. It is noteworthy that using an IP-MS/MS method with a detection limit of 78 pg/ml, we failed to detect pS129 in the CSF from PD patients or HC (unpublished data). The great variability of pS129 levels identified in the CSF of patients suffering from different synucleinopathies ranging from no detection^53^ to values ranging from pg/ml to μg/ml scale [(1.84 ± 0.71 to 265 (208–296) pg/ml; (7.14 ± 9.19) μg/ml]^49,50,77–80,128–131^, combined with the failure of several groups to replicate some of these studies and lack of validation by antibody-independent methods, has precluded the use of pS129 levels as a reliable biomarker.

#### pY39

In addition to pS129, recent studies have also explored the potential of assessing pY39 aSyn levels as a potential CSF biomarker. These studies were motivated by prior studies demonstrating that the levels of the activated form of c-Abl kinase, which phosphorylates aSyn efficiently at Y39^135^, are increased in the striatum and SN in PD brains^136^ and that an inhibitor of c-Abl increases aSyn clearance and is neuroprotective in preclinical models of PD^137–140^. Furthermore, in a small nonrandomized study of twelve PD patients, treatment with the c-Abl inhibitor nilotinib was reported to lead to an improvement in motor and cognitive symptoms^141^. However, a recent Phase 2 study reported that nilotinib showed no effect on symptoms or disease progression in either moderate or advanced PD.

To determine whether pY39 could serve as a biomarker differentiating PD patients from HC or patients with other synucleinopathies, Na et al.^142^ developed a targeted MS approach for the quantification of pY39 in the CSF. The assay was used to assess pY39 in CSF from a small cohort of PD patients (*n* = 4) and HC (*n* = 4) and showed no significant differences in pY39 aSyn levels between the two groups. However, the ratio of pY39 to Y39 was significantly increased in PD patients^142^. It is noteworthy that pY39 levels in the CSF are very low, in the range of 1.53–4.98 attomoles/ml^142^ (Fig. 4a, c). Therefore, the presence of minute amounts of unlabeled pY39 peptide standards could complicate the accurate estimation of its levels. Therefore, for targeted proteomics, it is of critical importance that the heavy standard peptide is spiked in at low quantities for reliable retention time and MS identification of the heavy peptide standard. The heavy/light ratio should be well above 1% to rule out any analytical bias and discard light isotope contamination derived from the standard and ultimately allow accurate quantification of the peptide/PTM of interest. Therefore, the reported levels of pY39 should be interpreted with caution^142^, and further studies in larger cohorts are needed to confirm the recent findings and to validate the assays used to detect and quantify aSyn pY39 as a potential PD biomarker.

### aSyn in the blood (plasma and serum)

**Fig. 5:**
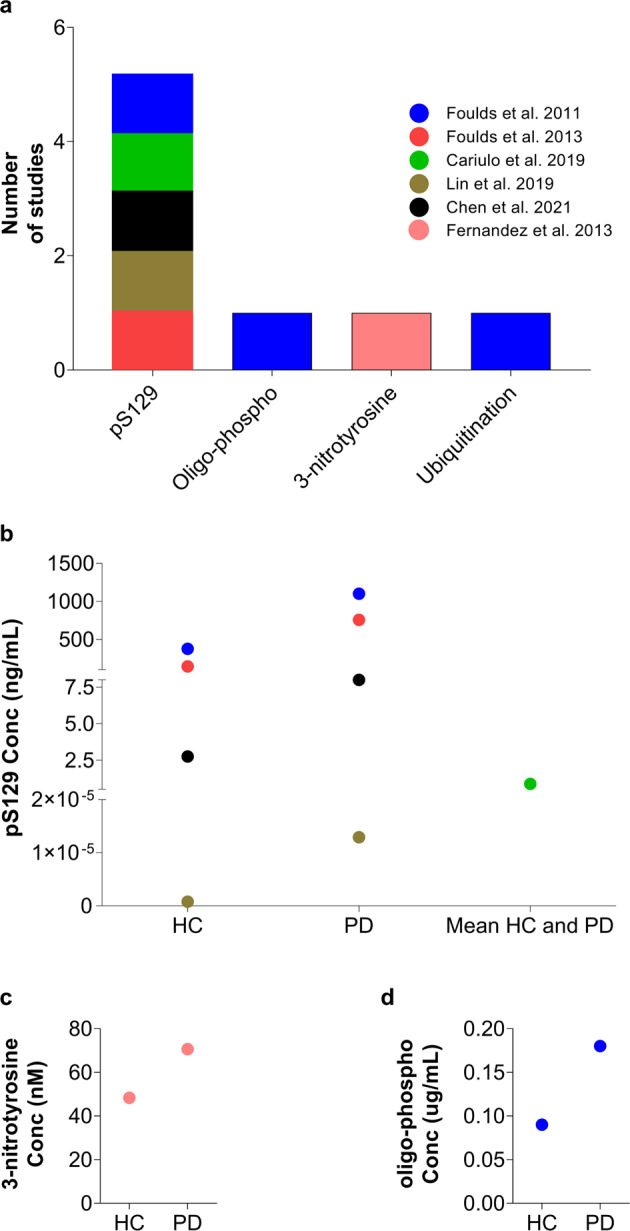
aSyn PTMs in Plasma/Serum. **a** Numbers of studies that reported the detection of aSyn PTMs in plasma/serum. **b** Variability of pS129 concentration between HC and PD in the different studies. Each dot represents the mean concentration of pS129 aSyn reported in each study. The mean of HC and PD is displayed for Cariulo et al.^53^ because only one pS129 level was provided in that study. Other aSyn PTMs, namely, **c** serum 3-nitrotyrosine^145^ and **d** plasma oligo-phosphorylation^54^, were also reported, but only in a single study. Ubiquitinated aSyn levels are not shown because this modification was assessed and quantified only by WB. The legend/color code in **a** corresponds also to **b**, **c** and **d**.

Human blood represents an alternative biological fluid that can be easily obtained using minimally invasive methods. A few studies have reported the identification and quantification of total plasma aSyn, which has been reported in varying concentrations on the scale of thousands of pg/ml (i.e., 3600 to 1,777,100 ± 3,609,600 pg/ml)^53–59^ (Table 2). The presence of posttranslationally modified forms of aSyn in plasma, particularly pS129, has also been investigated (Fig. 5a) and was reported to vary significantly across different studies (0.8 ± 0.6 fg/ml to 12.9 ± 8.7 fg/ml^143^ and 878.5 ± 317.4 pg/ml to 756.8 ± 2419.9 ng/ml^53,55^) (Fig. 5b and Table 1).

The levels of total, pS129 and oligomeric aSyn levels in different blood-derived components, including plasma and serum, have also been assessed as potential diagnostic and prognostic biomarkers of PD (reviewed in refs. ^73,75^). Using PTM-specific antibodies, Foulds et al.^54^ reported that aSyn pS129 levels were slightly increased in the plasma of PD patients compared to HC, but no significant differences were reported at the levels of oligomeric pS129 aSyn between the two groups (Fig. 5c). Remarkably, using the rabbit polyclonal anti-ubiquitin antibody FL‐76 (Santa Cruz Biotechnology), they described the detection of mono- and polyubiquitinated aSyn species in plasma; however, the ubiquitination sites and chain lengths were not defined. In a follow-up study, the authors showed that pS129 levels remain unchanged over a span of 20 years after the initial manifestations of PD symptoms^55^. However, subsequent studies showed an increase in pS129 levels in the plasma of PD patients^53,55,143^, suggesting that plasma pS129 may be a valuable PD biomarker. Interestingly, in a recent study, the plasma pS129 levels did not correlate with cognitive decline^143^ but were associated with motor severity and disease progression (i.e., increasing levels of pS129 over time) in a follow-up study of 3.5 ± 2.1 years^143^.

Recently, Chen et al.^144^ developed a novel approach using a modified PSPWB coupled to an immunoassay and a label-free technique for quantifying pS129 aSyn in diluted human serum and assessing its suitability as a diagnostic biomarker for PD. They reported that pS129 could be detected in diluted human serum human plasma in the range of 0.5 to 5 ng/ml in HC, whereas in PD, its levels ranged from 4 to 12 ng/ml. Interestingly, when comparing the levels of pS129 aSyn levels between PD patients and HC, they observed a substantial area under the curve (AUC) value (0.92), indicating that pS129 aSyn in diluted serum could be a potential PD biomarker. However, this study relied on a small sample size (10 PD patients and 11 HC); thus, further studies are required to validate these findings.

In a brief report published in 2013, Fernandez et al.^145^ described that nitration of aSyn could be detected in serum but not in CSF samples and suggested higher levels of nitration at Tyr125/136 residues and lower levels of nitration at Y39 in PD patients than in healthy controls (Fig. 5c). Moreover, the ratio of Tyr125/136 to Tyr39 aSyn was higher in early PD patients than in controls or advanced PD patients. However, no subsequent studies have confirmed or validated these findings or the antibodies used by the authors.

### aSyn in the RBCs

RBCs, or erythrocytes, are an additional blood-derived component that has been extensively explored for potential biomarkers. This compartment is the most abundant cellular fraction of human blood and is recognized as the primary source of aSyn in the blood^60^. Initial studies by Barbour et al. reported that ~99% of blood aSyn species are derived from RBCs, where its concentration is 26,200 ± 3000 ng/ml^60^. Recently, this was confirmed, to some extent, by two independent studies based on quantitative MS/MS approaches^61,62^, where aSyn was reported to be among the 20 most abundant proteins in RBCs^61^, and its concentration in RBCs was later reported to be in the range of 40 μg/ml^62^ (Table 2).

Vincent-Miranda et al. reported the detection of several posttranslationally modified forms of aSyn in RBCs, including phosphorylation (Y125), nitration (Y39), glycation and SUMOylation^146^. They reported increased levels of pY125, nY39, and glycated aSyn in the RBCs of PD patients compared to controls. In contrast, SUMOylated aSyn levels were decreased in PD patients compared to HC. Furthermore, the authors failed to detect ubiquitinated aSyn in these samples^146^. This study was based on the detection and quantification of modified aSyn forms through immunoblotting analysis (dot blots) using a selected set of antibodies against aSyn PTMs, mainly a single antibody against each selected PTM. However, in this study, it was not clear whether several antibodies were screened before the selection of antibodies against PTMs, and the main findings were not validated by independent techniques such as MS/MS. In addition, the method used in this study (comparison of dot blots) is not considered a robust, precise and sensitive quantitative assay for the assessment of PTM concentrations in RBCs and could lead to inconclusive evidence regarding their potential value as biomarkers of PD.

Because RBCs are considered the major source of aSyn in the blood^60^, different studies were conducted with the main aim of purifying aSyn from RBCs through extensive protocols based on multiple chromatography steps^147,148^. Using immunoblotting analysis and MS-based approaches, it has been shown that full-length aSyn is the predominant species in RBCs. In addition, a truncated form of aSyn was consistently detected by WB in samples from healthy donors. However, to the best of our knowledge, the precise sequence of this truncated form has not been mapped, and there have been no studies to determine whether its levels change in synucleinopathies or during disease progression.

**Fig. 6:**
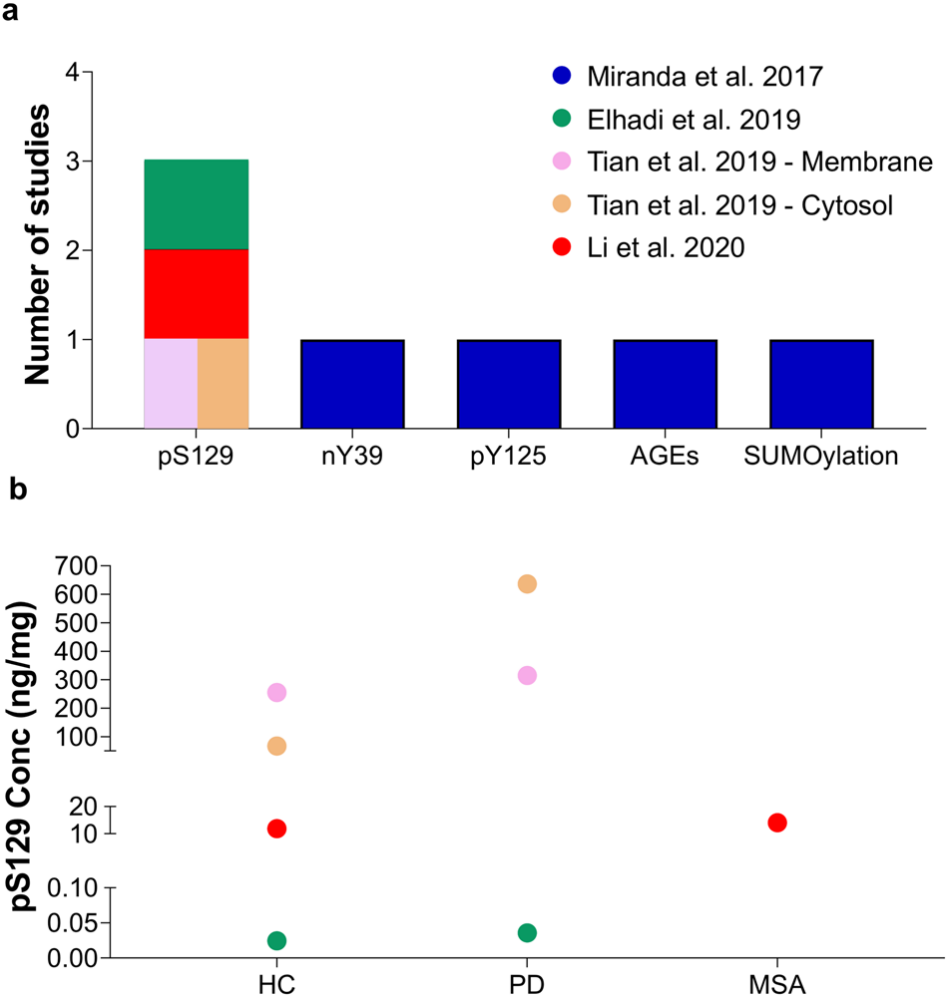
aSyn PTMs in RBCs. **a** Number of studies that reported the identification of different aSyn PTMs in RBCs. **b** Variability of pS129 levels across the different studies comparing PD to HC or to patients with other synucleinopathies such as MSA. In this graph, it is also depicted that RBC pS129 levels differ between the membrane and cytosol fractions^149^. Each dot represents the mean levels of pS129 aSyn reported in each study. The levels of aSyn PTMs such as nY39, pY125, AGEs or SUMOylation are not depicted in the figure because they were assessed only by dot and Western blotting analysis^146^; no assay for obtaining an “absolute” quantification was carried out. The legend/color code in **a** corresponds also to **b**.

In addition to the abovementioned aSyn PTMs in RBCs, pS129 levels have also been identified and detected in the range of 24.48 ± 7.6 to 636,050 ± 6030 pg/mg^149–151^ (Fig. 6a, b and Table 1). The levels of pS129 in RBC subcellular fractions were reported to be increased significantly in the cytosolic fraction and to a lesser degree in the membrane fraction of samples from PD patients compared to controls^149^. Elevated pS129 levels in RBCs were also reported in patients with purely motor PD compared to HC, whereas lower levels of pS129 were observed in PD with cognitive impairment than in purely motor PD^150^. In another study using WB and ELISA, Li et al.^151^ reported significantly higher pS129 aSyn levels in MSA patients than in HC. The authors also described significant differences between two subgroups of MSA patients [parkinsonian (MSA-P) and cerebellar (MSA-C)]. MSA-P patients displayed higher pS129 values than MSA-C patients^151^.

The increased levels of aSyn in RBCs compared to the other blood components and biological fluids could explain why it is easier to detect higher levels of modified forms of aSyn and suggest that RBCs may be a valuable source of aSyn biomarkers for the diagnosis and prognosis of PD. However, more systematic studies are needed to profile and quantify the levels of the different aSyn species in RBCs from large cohorts using a combination of robust unbiased and targeted proteomic approaches^62,152^.

### aSyn PTMs in saliva and tears

In addition to CSF and blood-derived fluids, saliva and tears have also been investigated as potential sources of biomarkers for PD because both types of fluids can be collected via a noninvasive method and are free from blood contamination. To the best of our knowledge, no studies have reported the identification and quantification of posttranslationally modified aSyn in saliva or tears, which could be attributable to the low levels of aSyn in these fluids compared to CSF, plasma and RBCs [concentrations in saliva are 7.104 ± 5.122 to 314.03 ± 435.90 pg/ml^64–66^ or even on a ng/mL scale (i.e., 159.4 ± 61.6 to 229.9 ± 64 ng/ml)^67^; concentrations in tears range from 32.02 to 361.16 pg/mg^68,69^].

### Oligomeric aSyn

The lack of correlation between LB pathology and PD development or severity, combined with accumulating evidence pointing toward the increased toxicity of oligomeric forms of aSyn, inspired efforts to search for oligomeric aSyn species in biological fluids as well as to assess their potential as biomarkers for PD and other synucleinopathies. Over the past two decades, several groups have reported the detection of aSyn oligomers in CSF^52,81,82,134^, blood constituents (plasma and RBCs)^82,153–157^ and other biofluids such as saliva^65,66,158^, relying primarily on immunoassays such as ELISA.

In CSF, oligomeric aSyn levels were mainly reported to be increased in PD compared to controls^51,52,81,82,125,130,134^. The diagnostic potential of aSyn oligomeric species was assessed in a meta-analysis report considering the levels of this aSyn species from different studies. In this report, the authors demonstrated that the sensitivity and specificity of assays for oligomeric aSyn forms remain unsatisfactory and not adequate to support clinical decision-making^51^. Nevertheless, the ratio of oligomeric aSyn to total aSyn levels in CSF^81,128,133,134^ displayed an improved diagnostic accuracy over utilizing the levels of the former alone, implying that this ratio is potentially a reliable PD biomarker.

The association between oligomeric aSyn levels and disease progression was also evaluated in different follow-up studies. Majbour et al.^78^ reported the association of oligomeric aSyn levels with PD severity and progression. This finding was further confirmed in subsequent studies by the same group^79,80,132^, suggesting that oligomeric aSyn species could be utilized as prognostic PD biomarkers. On the other hand, Murakami et al.^159^ observed no association between oligomeric aSyn levels and disease progression.

Additionally, aSyn oligomer levels have been explored in blood constituents. In 2006, El-Agnaf et al. reported that plasma aSyn oligomeric levels were significantly higher in PD patients than in control subjects^153^. These findings were further confirmed in consequent studies from different groups reporting an increase in aSyn oligomeric levels in plasma^154^ and red blood cells^156,160^. However, in other reports comparing the levels of blood aSyn oligomeric levels between PD and control individuals, no significant difference was identified^82,155,157^. Interestingly, in a recent report, Tian et al., using an ECL immunoassay, reported that oligomeric aSyn levels were significantly increased in the membrane fraction of RBCs in PD patients compared to control subjects, while no significant changes were identified in the cytosolic fraction of RBCs^149^. Furthermore, the levels of aSyn of oligomeric species were assessed in studies of salivary biomarkers, showing an increase in oligomeric aSyn levels in addition to a higher ratio of aSyn (oligo) to aSyn (total) in PD patients than in controls^65,66,158^. Nonetheless, these findings need to be confirmed in further studies using larger cohorts and by independent laboratories.

#### Posttranslationally modified oligomers

A review of the literature revealed a lack of studies evaluating and determining the levels of modified aSyn oligomeric and/or aggregated species. Indeed, to the best of our knowledge, only two studies, published by the same group, attempted to quantify the levels of aggregated phosphorylated species of aSyn in CSF and plasma (see sections: pS129 in CSF and aSyn PTMs in plasma/serum; Table 1). In both studies, the quantification of oligomeric phosphorylated aSyn species was performed using an in-house ELISA that relied on a commercially available antibody (anti-pS129 from Epitomics) as the capture and an in-house biotinylated p129 antibody as the detector. Outside this work, the presence of oligomeric phosphorylated species and their value as a biomarker have not been further explored. Moreover, the in-house ELISA used for the quantification of oligomeric-phosphorylated species could not differentiate between the different forms of aSyn, i.e., oligomers and fibrils^49,54^. Therefore, it remains unknown whether the aggregated phosphorylated species reported in CSF and plasma represent oligomers, fibrils or other aggregated forms of the aSyn protein.

One of the major challenges associated with measuring oligomeric forms of aSyn is that we have no insight into the biochemical and structural properties of native oligomeric aSyn. Because of their dynamic and unstable nature compared to the highly stable and protease-resistant fibril forms of the protein, some types of aSyn oligomers can be more susceptible to dissociate as recently reported by Luth et al.^161^. Therefore, artificial oligomers, usually one type, are used as calibrants, and in most cases, a chemically modified oligomeric form of the protein is used. The extent to which these chemical modifications affect the binding of the calibrants to antibodies or the ability to measure their concentration accurately has not been assessed. This, combined with the diversity of oligomer calibrants used and differences in the purity of the calibrants (i.e., the presence of other aSyn species (monomers or fibrils)), could also contribute to the considerable variation in absolute levels of aSyn oligomers measured by the different assays across different laboratories. Finally, it is important to stress that no current assay used to measure oligomers distinguishes between oligomers and fibrils;^49,54^ thus, labeling these assays as oligomer assays is misleading. Until we have tools that differentiate between oligomers and other aggregated forms of aSyn, we should use the terms aSyn aggregates instead of aSyn oligomers and aSyn aggregate assays instead of aSyn oligomeric assays. Another major limitation in the measurement of aSyn oligomeric species as a diagnostic and prognostic marker for PD and other synucleinopathies is the scarcity of commercially available kits. This drawback drove the PD research community to develop different in-house assays. Despite the efforts in developing assays to monitor and quantify oligomers, comprehensive characterization of the employed calibrants and antibodies was not always carried out or made available, including independent validation of the biotinylated and in-house antibodies and the aggregate-species specificity of the antibodies (i.e., specificity for aSyn oligomers vs. other aggregated species) in addition to the purity of the calibrants (i.e., characterization of oligomers or aggregated forms by EM)^79,80,132^.

Therefore, it is of paramount importance to (1) conduct further studies using a well-characterized and validated set of antibodies against the aggregation states of the aSyn protein^162^ utilizing not only recombinantly generated standards but also cellular models to mimic physiological conditions; (2) extensively share data regarding the new antibodies and include the validation data in the reports; (3) openly communicate the characterization and validation data of the newly generated antibodies in the published reports; and (4) use properly characterized calibrants, ensuring that the reported observations are against aSyn oligomers and no other aggregated species.

### Seeding-competent aSyn species

Sensitive and specific aSyn SAAs, also named in the literature as RT-QuIC and/or PMCA, have been developed and optimized to amplify and detect minute amounts of aggregated aSyn not only in CSF^35,85–88^ but also in peripheral tissues (e.g., skin or colon biopsies)^36,72,83^.

Initial studies attempted to validate the seeding activity of aSyn by comparing brain and CSF samples. Interestingly, the great majority of the studies using these assays with CSF samples displayed good performance (high accuracy, sensitivity and specificity) as a potential diagnostic tool for PD^163^. In one comparative study, two independent groups evaluating the same sample set using two different aSyn SAAs (PMCA and RT-QuIC) reported similar results, exhibiting high sensitivity and specificity values, reflected by an improved AUC (i.e., PMCA-AUC = 0.93 and RT-QuIC-AUC = 0.89) for the clinical diagnosis of PD^35^. Besides, when considering only the samples with consistent findings similar in both aSyn SAAs, the AUC increased to 0.94. Overall, the authors emphasized and confirmed the reproducibility of both assays and their accuracy/performance for PD diagnosis^35^. In a recent study, Russo et al. corroborated, to some extent, the reproducibility and potential of different aSyn SAAs as a diagnostic PD tool^87^. In more detail, the authors performed a blind comparative study that relied on aSyn SAAs developed from three independent research laboratories, and each laboratory used its selected material/reagents and methodologies. They reported that the aSyn SAAs exhibited concordant findings among the three labs, with small variations in sensitivity and specificity determined by each group^87^.

Over the past few years, the aSyn SAA assays have been increasingly used and refined to allow for differentiating between PD and other synucleinopathies. Fairfoul et al.^85^ compared a small cohort that comprised patients suffering from DLB, DLB with AD, AD with incidental LB, PD and HC. They reported that aSyn PMCA could identify controls and patients with tauopathy-related conditions with perfect specificity and that it had high sensitivity in discriminating the different synucleinopathy-related cohorts except for DLB with AD. Remarkably, several groups have corroborated and demonstrated similar results in discriminating PD from controls, highlighting the performance of aSyn SAAs as a potential diagnostic tool^35,72,83,84,87^.

Other studies have also compared the performance of aSyn SAAs in distinguishing PD from other synucleinopathies. Using aSyn SAAs, CSF aSyn strains could be discriminated^86,88,164^, suggesting that pathogenic aSyn species might display different conformations in different aSyn-related disorders. Moreover, these assays may play a key role in the preclinical identification of patients who may progress to PD^34,89^. In greater detail, Shahnawaz et al. demonstrated that the aSyn PMCA could discriminate PD and controls with high sensitivity and specificity and showed correlation with the severity of the disease. Interestingly, they also showed that aSyn PMCA could open new avenues in preclinical risk stratification, identifying patients who may develop PD^89^. This was further confirmed, to some extent, in recent reports that study idiopathic rapid-eye-movement sleep behavior disorder (iRBD)^86,89^.

In addition to studies focusing on aSyn SAAs in CSF, several other studies have sought to evaluate the performance of these assays using peripheral tissues (e.g., skin^36^, olfactory mucosa and colon biopsies^35,72,83–85^), also displaying promising results in terms of sensitivity and specificity. For more recent reviews on the development of aSyn SAA and their recent applications in biomarker discovery and characterization of different synucleinopathy cohorts, please refer to ref. ^163^.

Regardless of the high performance of aSyn SAAs as diagnostic tools, one of their major shortcomings is the lack of quantification of the aSyn aggregates, which consequently may hinder their application for monitoring disease progression. As kinetics parameters are normally used to measure aggregation rates, the quantification of aSyn aggregates when seeded with the biological specimen from PD and other synucleinopathy-derived patients remains to be determined. A recent study by Russo et al.^87^, comparing aSyn SAAs from three independent labs, showed some potential interesting associations of kinetic parameters with clinical data that could allow for monitoring disease progression and severity. However, the authors could not confirm the value of these assays to monitor disease severity/progression, as they identified inconclusive findings across all three aSyn SAAs/labs. Therefore, the authors suggested that quantitation may solely be feasible via multivariate analysis with power statistical analysis and appropriate normalization of the data from larger cohorts. Remarkably, a recent report showed that RT-QuIC may be useful to monitor MSA disease progression^86^. Poggiolini and collaborators confirmed the potential value of RT-QuIC as a diagnostic tool for PD as well as to distinguish MSA from PD. They also reported that the RT-QuIC quantitative parameters correlated with worse clinical MSA progression, but not with PD clinical scores. Furthermore, they demonstrated the potential of SAA as an early diagnostic tool, predicting iRBD patients who may develop synucleinopathy pathologies^86^. Overall, this study^86^ emphasizes that aSyn SAA may open new avenues for early clinical and intervention decision-making along with monitoring MSA disease progression. Nonetheless, more studies with larger sample sizes encompassing MSA but also other synucleinopathy subgroups and extended follow-up clinical data are required to establish whether aSyn SAA can be used as both diagnosis and prognosis tool.

Despite the high sensitivity and specificity shown for some assays using CSF and peripheral tissues, the invasive collection methods and considerable demands on the patient and clinician may limit their clinical implementation and application. Therefore, we recommend the application of these assays to blood components (e.g., whole blood, erythrocytes or plasma), saliva and tears. This could pave the way for noninvasive novel diagnostic assays that could be used to support early diagnosis, patient recruitment and stratification for clinical trials; to assess target engagement in clinical trials of aSyn targeting therapies; or to monitor disease progression.

### aSyn in exosomes/extracellular vesicles in body fluids as biomarkers of PD

The cell-to-cell propagation of aSyn has been implicated as a central process in aSyn pathology spreading and PD progression, although the exact mechanisms regulating this process remain a subject of active research^165,166^. Given their presence in biological fluids and their important role in cell-cell communication, extracellular vesicles (EVs) have emerged as possible candidates for regulating the cell-to-cell transfer of aSyn and pathology spreading. Furthermore, several proteins involved in proteinopathies (aSyn, amyloid-beta and tau) have also been found in EVs^167–169^. Therefore, several studies pursued EVs (predominantly exosomes)-derived aSyn as a potential diagnostic biomarker for PD.

Exosomes are EVs produced by different types of cells, have diameters ranging from 50 to 100 nm, and contain a complex mixture of molecules, including proteins, lipids, and nucleic acids. Their secretion, transfer from one cell to another, and ability to cross the blood-brain barrier and move between the brain and systemic circulation make EVs central players in cell-to-cell communication in health and disease^170^. They can transport and spread both physiological and pathological molecules, including toxic and seeding competent protein aggregates such as aSyn^171^. For example, microglial exosomes facilitate aSyn cell-to-cell transfer^172^ and pathology formation. Interaction between aSyn and cell-derived exosomes has also been reported to accelerate aSyn aggregation in vitro^173^.

The availability of different protocols to isolate EVs from biological fluids (CSF, plasma, urine, and saliva) prompted studies to evaluate their potential as diagnostic biomarkers for PD and other NDDs. Several studies have reported changes in aSyn levels in CSF^174^, plasma/serum^59,175–181^, and salivary exosomes in PD and synucleinopathy patients^182^. Other studies reported that exosomal aSyn species can seed aSyn aggregation in vitro and pathology formation and spreading in vivo. For example, Stuendl et al. reported lower levels of EV-bound aSyn in the CSF from PD and showed that EVs aSyn from the CSF of PD or DLB contains seeding-competent aSyn species^174^.

aSyn is very abundant in RBCs and RBCs-derived aSyn could be a major source of aSyn contamination in the CSF^59,122^. Therefore, different strategies have been employed to specifically isolate and quantify CNS and brain-derived EVs, including the use of antibodies directed against the neural L1 cell adhesion molecule (L1CAM) as well as to SNAP25, EAAT1, OMG)^179,183,184^. Initial studies by Shi et al. reported the isolation of the CNS-specific EVs from plasma using a magnetic bead-based capture assay that pull-down L1CAM-derived EVs and showed that aSyn is present in these EVs and at higher levels in PD patients, compared to the control cohort. They also reported a significant but weak correlation between exosomal aSyn levels and disease severity^59^. Several other studies also showed increased levels of EV-associated aSyn in plasma and serum of PD patients^175–180^. In contrast, Si et al. reported decreased levels of serum EV-derived aSyn in PD patients^181^.

In a recent report, it has been suggested that the ratio of aSyn levels in potential oligodendroglial exosomes compared to neuronal exosomes enables to differentiate betweeen PD and MSA patients^180^. The value of plasma neuronal exosomes as a prognostic marker for PD progression was also evaluated in a follow-up study of 22 months. It was shown that EVS-derived aSyn levels were associated with a higher risk of motor symptoms^177^, but further studies are needed to validate these findings.

Subsequent studies investigated changes in the levels of pathological aSyn species (oligomers/fibrils, insoluble, proteinase K resistant aSyn, and pS129 aSyn) in relation to total aSyn in plasma/serum exosomes from PD patients and HC^177,181,183,185^. Using two exosome purification strategies and a battery of assays such as Electron Microscopy, nanoparticle tracking analysis and WB, Zheng et al. identified oligomeric and phosphorylated aSyn species inside but also on the membrane surface of plasma exosomes^185^. They reported lower levels of total aSyn and higher values for the ratios of aSyn oligomer/total aSyn and aSyn/total aSyn or pS129 pS129 in the plasma exosomes of PD patients. Higher levels of oligomeric aSyn EVs (oligomeric aSyn/total aSyn) were also seen in salivary EVs^185^. However, there was no correlation between these values and disease severity. It has been also shown that the levels of aSyn in L1CAM-positive EVs are higher in individuals with REM sleep behavior disorders^177,178^, who are known to be at higher risk of developing PD. Interestingly, the levels remain high in individuals who progress to develop PD^177^. Very limited studies have been carried out to investigate changes in the levels of oligomeric aSyn species in neuron-derived EVs. In one study, lower levels of oligomeric aSyn levels were reported in PD patients, with differences seen between PD patients with and without tremor symptoms^181^, whereas other studies reported higher levels^177,183^. Other studies have also investigated the correlation between pS129 aSyn levels and PD. Jiang et al. reported increased neuronal exosomal pS129 aSyn levels in a subgroup of PD patients, in the absence of a correlation with disease severity^178^. In another study, unmodified and pS129 aSyn species were found on the membrane surface and inside exosomes and exhibited reduced solubility after PK treatment^185^.

Studies of EVs carrying aSyn were predominantly focused on CSF and blood-derived samples, however, EVs have also been evaluated in body fluids such as urine and saliva^182,186,187^. EVs-derived aSyn was identified in saliva^182,187^, but not in urine^186^. The salivary levels of aSyn oligomers along with the ratio of aSyn oligo/aSyn total were reported to be higher in PD when compared with controls^182,187^. However, the levels of these aSyn species did not show any association with age and disease duration^182,187^.

Although several studies have shown that differences in the levels of aSyn species in EVs could differentiate PD patients from HC, such differences are not robust enough to allow for monitoring disease progression. It has been suggested that this could be because the EVs measured include a combination of both brain- and peripheral nerves-derived EVs^182,183^. Further longitudinal and follow-up studies in larger cohorts are needed to improve the sensitivity and specificity of EVs aSyn species and to validate their potential as biomarkers to monitor disease progression or differentiate between PD and other synucleinopathies. Furthermore, a better understanding of the mechanisms responsible for the secretion and clearance of Evs aSyn and the role of specific EVs aSyn species in disease development and/or progression could pave the way for achieving this goal. It is plausible that exosomal secretion of specific forms of aSyn represents a protective mechanism aimed at lowering the levels of intracellular aSyn. In contrast, secretion of aggregated forms may represent a toxic mechanism by facilitating the spreading of toxic aSyn species and pathology in the brain. Finally, recent studies suggest that the incorporation of other protein biomarkers, including DJ-1 and LRRK2, but also blood proteins such as clusterin, apolipoprotein A1, and disease-triggering miRNAs and mRNAs^176,182,186–188^ could improve the diagnostic potential of EVs. For recent and detailed reviews on EVs formation, function and diagnosis and prognosis utility, please see refs. ^167–169,188–190^.

### Outlook

#### Clinical PD research: challenges and recommendations

Our review of the literature on the presence of aSyn species in biological fluids exposed several challenges that hinder progress in aSyn biomarker discovery and validation: (1) the lack of standardized guidelines for sample collection and handling (i.e., preanalytical and analytical confounding factors) and patient selection criteria; (2) the nature of the methods/assays that focus only on detecting a single aSyn proteoform rather than embracing the PTM complexity; (3) the use of nonspecific antibodies or antibodies that have not been well-characterized and validated using the appropriate protein standards; and (4) the usage of poorly characterized or impure protein standards. Therefore, in the following section, we expand on discussing these challenges and provide recommendations to address them as an essential step to enable (in the near future) systematic assessment of the potential of aSyn (total, PTMs or aggregated forms or their combination) as biomarkers for (1) early detection and monitoring of disease progression; (2) patient stratification and (3) evaluating the efficacy of novel therapies.

##### Sample size and diversity

**Fig. 7:**
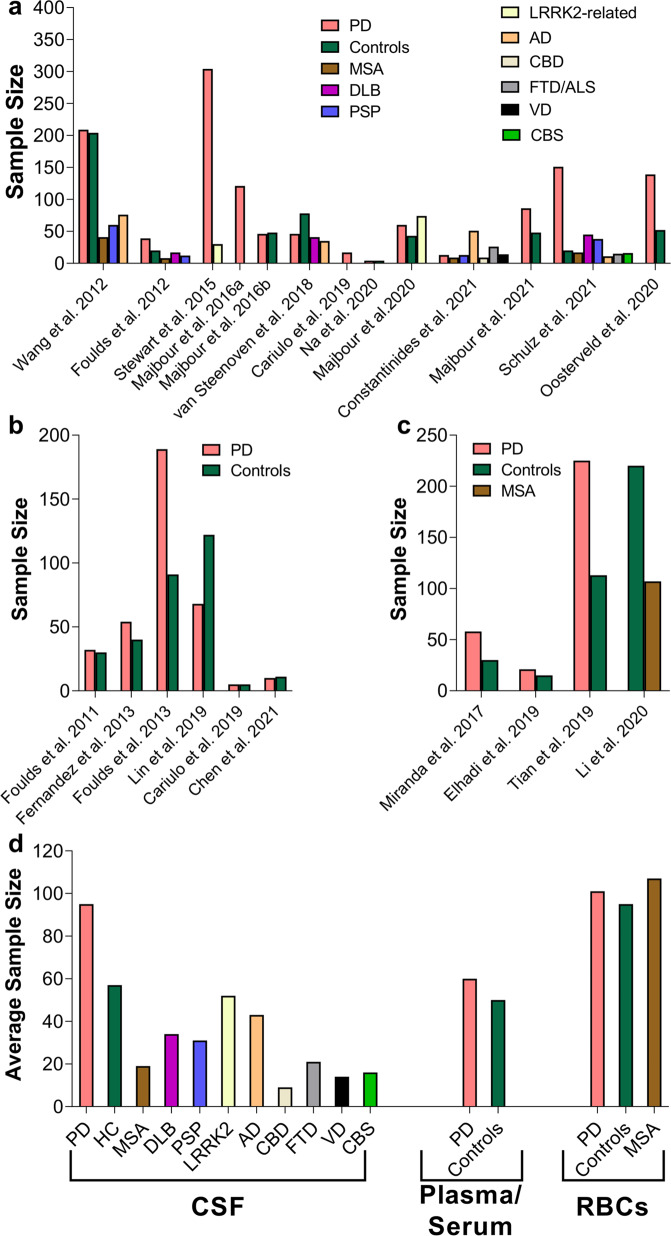
Sample sizes and averages of the different disease groups in studies aimed at quantifying aSyn PTMs in different body fluids. Sample sizes in **a** CSF; **b** plasma/serum; and **c** RBCs. **d** Average sample size of different diagnostic cohorts in different body fluids (i.e., CSF, plasma/serum and RBCs). The average sample sizes of the LRRK2-related, CBD, FTD, VD and CBS groups in CSF studies, as well as the MSA group in RBC studies, represent absolute values since these disease groups were evaluated in only a single study each.

Our review of the literature also revealed that the vast majority of the studies on aSyn PTMs as biomarkers relied on small sample sizes. The small sample size in PD research led to a poor assessment of variability between patients and experimental reproducibility across different laboratories (Fig. 7a–c).

Particularly, in the CSF studies, aSyn PTMs have mainly been assessed in HC and PD (Fig. 7a) with patient cohorts ranging in size from 8 to 304 individuals, with only a handful of studies using sample sizes of >100 individuals. Interestingly, in aSyn PTM studies, when considering the average size of the cohorts, an average of 60 controls and 90 PD patients were observed (Fig. 7c). Among the studies investigating CSF aSyn PTMs as potential biomarkers, only a few studies evaluated their levels using samples derived from patients suffering from synucleinopathies other than PD (Table 1 and Figs. 4b and 7a)^49,50,77,80,128,129^. The average sample size decreases dramatically in studies on other synucleinopathies and neurodegenerative disorders (e.g., MSA: 20; DLB: 30; PSP: 30) (Fig. 7c). Moreover, only a single study included patients suffering from CBD, FTD and VD.

**Fig. 8:**
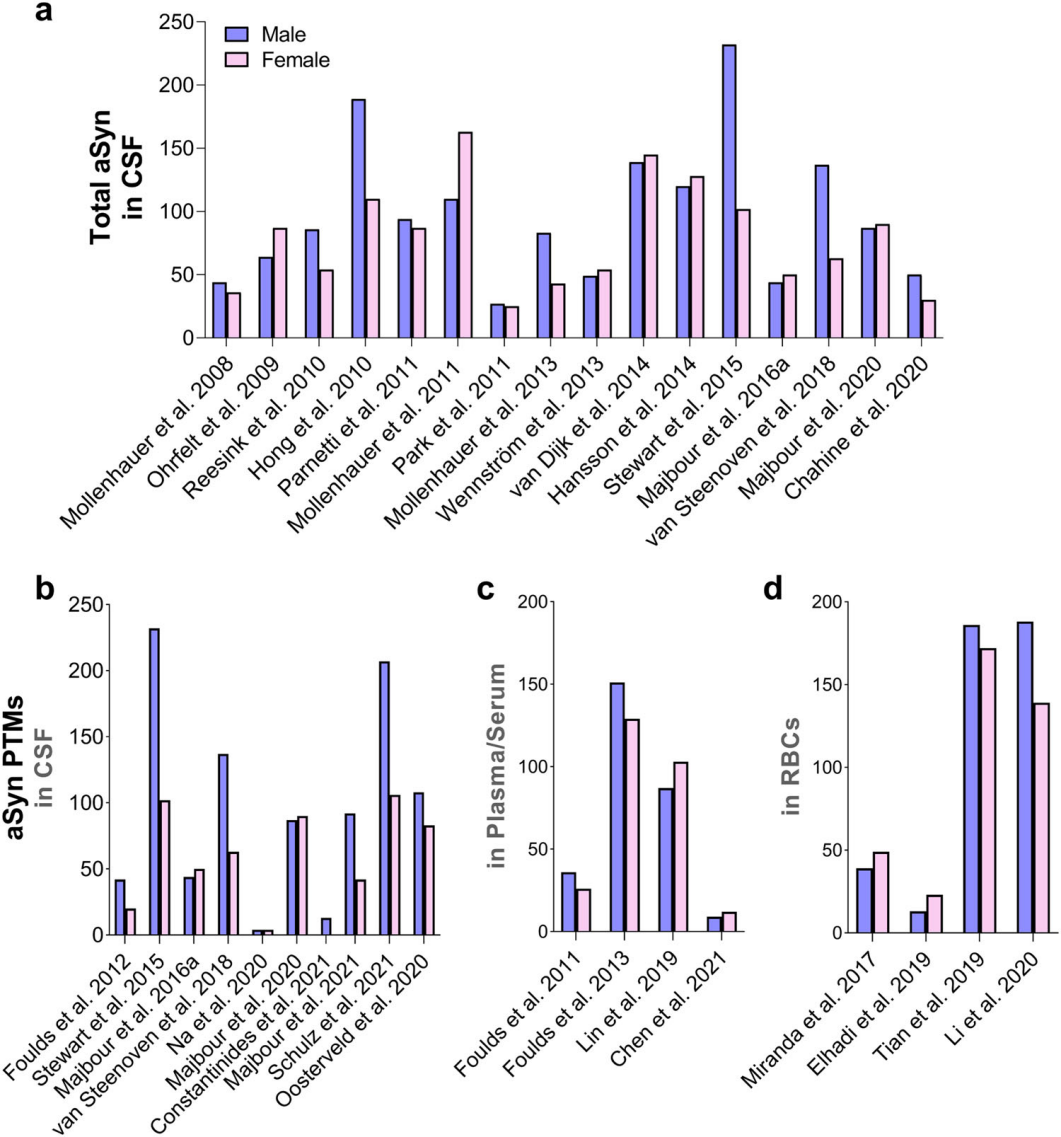
Gender representation across the different aSyn-biomarker studies. Male vs. female representation in studies that aimed to measure the levels of **a** total aSyn in CSF and aSyn PTMs in **b** CSF, **c** plasma/serum and **d** RBCs.

Remarkably, studies focusing on aSyn PTMs in blood constituents (i.e., plasma/serum and red blood cells) have mainly compared PD with controls^53–55,143,146,149,150,191^ (Fig. 7b, c) and relied mainly on less sensitive biochemical techniques such as ELISA. To the best of our knowledge, aSyn PTMs in plasma/serum studies have been evaluated solely in controls and PD patients with sample sizes ranging from 5 to 189 individuals (Fig. 7b). However, when all the studies are considered, the average sample size is 60 HC and 70 PD patients (Fig. 7d). Only in one study was the number of PD patients above the average sample size (*n* = 189)^55^. As in the plasma/serum studies, the levels of aSyn PTMs in erythrocytes were assessed in relatively small cohorts ranging in size from 21 to 225 subjects (Fig. 7c), with a mean cohort size of 90 HC and 100 PD individuals. One single study assessed the levels of pS129 aSyn in erythrocytes in other synucleinopathies, i.e., a comparison of 107 MSA patients with 220 controls^151^ (Fig. 7c, d). Additionally, regarding gender representation, most of the studies present gender bias, with male patients being overrepresented (Fig. 8). It is also important to stress that most of the studies have been carried out on white populations, encompassing American, European and Asian, while underrepresented groups from other ethnicities such as African Americans, Africans or middle east populations remain understudied^192–194^.

Altogether, the small sample sizes may be associated with different drawbacks of current approaches, such as (1) the invasive nature of CSF collection; (2) the nature of the patients’ demographic data (e.g., elderly and nonautonomous); (3) difficulties in the accessibility to the specialist centers that are mainly in urban areas; (4) low incentives for participation in the studies; and (5) the requirement of multiple sample donations.

Nonetheless, because of the heterogeneity of PD and other synucleinopathies, multicenter biomarker studies with large sample sizes and appropriate patient selection (e.g., age, gender, stages of the disease, patient clinical manifestations, lifestyle or comorbidities) encompassing PD and other synucleinopathy and neurodegenerative groups (e.g., MSA, DLB, PDD, PSP, AD) are needed. The large sample sets would allow the generation of a discovery cohort and independent validation cohorts with the appropriate sample sizes to enable rigorous statistical analysis and validation of reliable single and/or panels of synucleinopathy-related biomarkers. Thus, methodological mapping of aSyn species coupled with multicenter cohort studies of specific patient groups is necessary to allow systematic assessment of the potential of aSyn species as diagnostic biomarkers and to gain additional insights into the pathophysiology and PD and synucleinopathies. These studies will be facilitated by access to different types of biofluids and biopsies from the same patients and control cases and access to biofluids from cases with autopsy-confirmed diagnoses.

##### Sample collection

The great majority of the samples used in the published studies were collected under conditions that did not account for the reversibility of aSyn PTMs and were not optimized to ensure the preservation of the diversity of aSyn species in biological fluids during sample collection and handling. Previous studies to standardize sample collection focused primarily on preanalytical confounding cofactors^122,195,196^ such as (1) temperature at which the samples were collected; (2) type of collection tubes; (3) time between sample collection and sample storage; (4) multiple tube transfers (aliquots) and freeze-thaw cycles; (5) usage of nonionic detergents; and (6) collection of samples from various disease groups at different periods. When PTMs were considered, the guidelines for sample collection and standardization focused primarily on preserving serine/threonine phosphorylation. Therefore, standardization and validation of sample collection and handling techniques that both preserve the chemical integrity of the aSyn proteome (i.e., prevent aSyn degradation) and preserve the diversity of PTMs are needed. Each particular aSyn biomarker needs to be assessed via carefully conducted studies of preanalytical variables such as stability of the species during the sample collection procedure, storage, freeze/thaw effects, etc. A single set of SOPs is unlikely to be useful for preserving all aSyn species.

##### Modified aSyn protein standards

Initially, researchers in the field did not have access to pure protein standards of site-specifically modified aSyn proteins. Therefore, many preceding studies, aiming to quantify pS129, used aSyn protein standards that were generated by incubating recombinant aSyn with kinases that were later shown to phosphorylate aSyn only partially at S129 (e.g., casein kinase II), thus resulting in impure protein standards^49,50,54,55,77,151^. In many of these studies, it is unclear whether the phosphorylated protein was purified or whether mixtures of unmodified and pS129 aSyn of unknown proportion were used as pS129 standards^49,54,55,77,151^.

The discovery of the Polo-like kinases (PLKs): PLK2 and PLK3, as kinases that efficiently and quantitatively phosphorylate aSyn in vitro and in cells, made the generation of pure pS129 protein standards possible^53^. Unfortunately, in many cases where PLK2 or PLK3 were used, the extent of aSyn S129 phosphorylation was not reported. Again, in many cases it was not described whether the pS129 aSyn proteins used were purified from the in vitro phosphorylation reaction mixture (Table 1). Furthermore, in the majority of published biomarker studies, the analytical data on the purity and characterization of the protein standards used were not presented. Such data should be included in future studies to demonstrate that the protein standards used are highly pure and free of aSyn truncations, oligomers or other aggregated forms of the protein.

Over the past 10 years, our group has pioneered the development of several semisynthetic strategies that allow for the site-specific introduction of single or multiple PTMs throughout the sequence of aSyn^197–203^. These advances have enabled us to generate highly pure and homogeneously modified forms of aSyn that encompass the great majority of aSyn species detected in the brain, CSF and blood. The availability of these reagents and other semisynthetic proteins has already been instrumental in facilitating the development of novel and more robust tools and assays for the detection and quantification of aSyn through (1) the production of homogenously and site-specifically modified aSyn protein standards bearing single or multiple PTMs and (2) the generation of PTM-targeting antibodies^53,135,199,201,204^. Notably, the main pS129 aSyn protein standards used currently by researchers in the PD research community were produced on a large scale (hundreds of milligrams) using protein semisynthetic strategies developed by our group^199^ and through close collaboration with a contract research organization funded by the Michael J. Fox Foundation, or MJFF (e.g., pS129 aSyn protein, which is now commercially available through MJFF and Proteos, Inc). Therefore, the methods for generating high-quality aSyn protein standards exist and should be used to support biomarker discovery and validation.

##### Antibodies

The vast majority of biomarker discovery studies relied on the usage of antibodies as the primary tool for detecting and quantifying the different aSyn PTMs in the brain, peripheral tissues and body fluids. Our review of this work revealed that (1) the available antibodies recognized only a limited number of aSyn PTMs (e.g., nitration, pS129). The two major reasons for targeting pS129 aSyn as a biomarker are as follows: (1) it is among the most common and abundant PTMs in the brain, and (2) the availability of a large number of antibodies against pS129 that were shown to reproducibly detect pS129 aSyn in the brain as well as in some biological fluids and peripheral tissues^16,49,54,55,72,78–80,92,100^. Several antibodies against pS129 aSyn have been developed and made commercially available (e.g., pSyn#64 from Fujifilm Wako; pS129 antibody from Epitomics, now acquired by Abcam (EP1536Y); anti-pS129 antibody (825701) from BioLegend)^49,53,143,150^. Other antibodies against pS129 were developed in house^50,77–80,128,129,132^ and are not directly accessible to the scientific community.

Interestingly, most of the antibodies against pS129 aSyn have not been validated against proper protein standards that account for confounding effects due to the presence of other proteins and co-occurring or multiple aSyn PTMs. This validation is of key importance given the clustering of multiple PTMs in different parts of the aSyn sequence. Indeed, it has been demonstrated that pS129 antibodies that show high cross-reactivity to other proteins^205–207^. Besides, our group has shown that the presence of multiple PTMs could, for example, interfere with the detection of pS129 by some pS129 antibodies^208^. In a more recent study, we demonstrated that the co-occurrence of pS129 with other disease-associated PTMs in the vicinity of S129, including phosphorylation at Y125 (pY125), nitration at Y125 (nY125) or C-terminal truncations at residues 133 and 135, dramatically decreases or abolishes pS129 detection by many pS129 antibodies^209^. Moreover, using immunoblotting analysis, we have also shown that these antibodies cross-react with other proteins whose molecular weights are similar to those of aggregated aSyn species present in the brain^209^. Although most pS129 antibodies reliably detect pS129 aSyn in pathological aggregates or soluble pS129 aSyn under overexpression conditions, they exhibit high cross-reactivity when assessing endogenous pS129 aSyn under physiological conditions. These findings underscore the critical importance of validating aSyn antibodies under physiologically relevant conditions using protein standards that account for the complexity and diversity of aSyn PTMs in the brain and biological fluids. Although a number of studies have investigated the specificity of pS129 aSyn antibodies, there are no published studies where the specificity and cross-reactivity of antibodies against other aSyn PTMs, such as nY39, pY39, and pS87, have been systematically investigated.

Regarding the detection of nitrated aSyn, despite the commercially availability of several antibodies targeting nitrated aSyn (nY39, 36-012 (Millipore); nitrated aSyn (nY125/136): nSYn12 (Millipore); nitrosylated aSyn (nY39): nSYn14 (Millipore)), only a handful of studies have reported the quantification of nitrated aSyn species in body fluids using these antibodies^146,191^. Furthermore, despite the detection of other aSyn PTMs such as ubiquitination, acetylation, SUMOylation, glutathionylation, and glycosylation in the brain^90^, reliable antibodies against these PTMs are still lacking.

In human brain tissues, the second most commonly observed modified aSyn species result from C- and N-terminal cleavages of aSyn, particularly C-terminally truncated species^96,97,99^. Only a handful of aSyn truncation-specific antibodies have been generated and used to study the presence of truncated aSyn species in pathological aggregates, e.g., Syn105 from Prothena (recognizes aSyn truncated at position 122) and aSyn-131 (recognizes aSyn truncated at position 119) and aSyn-134 (recognizes aSyn truncated at position 122) from Roche^210^. Despite their existence, they are not readily accessible to the scientific community. There are no antibodies that specifically recognize N-terminally truncated forms of aSyn.

The mapping and profiling of truncated aSyn fragments in the brain have been achieved primarily by WB analyses and more recently by MS approaches (Fig. 2)^16^. Interestingly, none of the body-fluid-based reports described in this review attempted to specifically detect or quantify truncated aSyn or evaluate whether their presence could interfere with accurate determination of aSyn. A recent comparative study demonstrated that three of the most commonly used immunoassays (ELISA) to measure total aSyn levels failed to detect the most disease-associated C-terminally truncated forms of aSyn. It showed that several N-terminal truncations and phosphorylation differentially influence aSyn detection and recovery by the three immunoassays^211^. Overall, the majority of published or commercially available immunoassays relied on antibodies that were not thoroughly validated, or the validation data were not reported.

Among the major limitations of the current assays (e.g., ELISA, WB) are: (1) Some of the antibodies used may detect different forms of aSyn and not only the target aSyn species of interest, thus leading to inconclusive or irreproducible measurements. (2) Body-fluid-based biomarker studies have mainly focused on the quantification of the most prominent aSyn PTM, pS129, which does not reflect the complexity of aSyn PTM patterns in biological fulids^49,54,79,128,129,132^. For example, neither the possible presence of aSyn truncations nor the potential disruption of antibody binding to aSyn due to the co-occurrence of multiple modifications^96,97,99^ was taken into consideration in the measurements of aSyn species in body fluids. This could lead to underestimation of aSyn levels and contribute to the variability of aSyn levels across different studies. (3) A large number of the total aSyn biomarker studies relied on antibodies and assays that are designed to detect and quantify a single specific aSyn form rather than evaluating the diversity of aSyn PTMs. In other biomarker studies designed to detect and quantify single aSyn PTMs, the potential impact of the co-occurrence of multiple PTMs in close proximity to the target PTM was not considered during the selection of antibodies or optimization of the assays. Therefore, we recommend prioritizing (1) the development of more specific aSyn antibodies; (2) the development of antibodies that can recognize the different aSyn PTMs, preferably with residue specificity and selectivity; (3) the characterization and validation of antibodies using a library of aSyn protein standards bearing the desired PTM, including proteins comprising multiple physiological and pathologically relevant PTMs; and (4) thorough investigation of the specificity and cross-reactivity of all antibodies in different experimental settings. It is worth considering validating all commercially available and in-house antibodies through independent third-party entities^212^, which could catalyze the progress of novel and improved antibody-based tools for accurately assessing/mapping aSyn proteoforms and ultimately establish them in clinical practice as diagnostic and prognostic biomarkers.

##### Methods for measuring protein concentration

Immunoassays and antibody-based assays are among the most used for the identification and validation of protein-based markers, such as aSyn. Interestingly, despite using similar antibodies or antibodies that target the same region of aSyn, studies using these immunoassays have reported significant differences in the concentration of aSyn (Figs. 3a, c, 4b–d, 5b–d and 6b). Several factors have been proposed to contribute to the large deviations in aSyn levels measured with different kits^76,122^. One additional factor is the lack of well-characterized protein standards. The protein standards provided by different vendors have different concentrations and quality and are produced using different sample preparation and handling procedures. This could also contribute to the large differences in the aSyn levels measured by researchers. Unfortunately, most of the commercial immunoassays do not provide information about the purity (chemical integrity and aggregation state) of the protein standards used in their kits or provide sufficient protein material to allow for independent validation of the concentration or purity of the standards by the users.

Protein concentration determination of the calibrant is of key importance for the quantification of biomarkers. Several methods have been used to assess protein amounts, such as Lowry’s method, the Bradford method, the BCA approach and amino acid analysis (AAA)^213–216^. Spectrophotometric and colorimetric approaches are characterized as sensitive and reproducible, but their measurements can be affected by the conformation of the protein (aggregation states)^214–216^, as well as by the presence of reducing agents or surfactants/detergents. On the other hand, AAA, which relies on protein hydrolysis, allows protein quantity and amino acid composition to be ascertained accurately. Thus, it is considered a gold standard method for determining an absolute and exact quantitative measure of the protein standard^162,213,217–219^. Moreover, this method is independent of any external protein standard curve, protein charge, protein state, or dye-binding percentage. Hence, we recommend using AAA for assessing the absolute quantification of protein standards that will be used as the reference material in the standard curve for different protein concentration assays, such as ELISA and IP-MS/MS.

##### Unbiased mapping of aSyn species

Although the assays used in aSyn biomarker research could support multiplexing, the majority of studies on aSyn PTMs in biological fluids have mainly focused on a single combination of antibodies (detector and capture antibodies), with a strong emphasis on the detection of one PTM at a time.

Given the lack of antibodies that cover the diversity of aSyn species and the scarcity of data on the role of different PTMs in the physiology and pathogenic properties of aSyn, we recommend revisiting mapping of the aSyn proteoforms in the brain and biological fluids using unbiased experimental approaches, such as MS. This would enable researchers to identify differences in the aSyn proteoform in individuals with synucleinopathies compared to HC, thus enabling the field to prioritize the development of new antibodies targeting disease/pathology-associated aSyn and other abundant PTMs, tools, reagents and assays for biomarker discovery and validation. Altogether, this would lead to the development of novel antibodies and more sensitive approaches (e.g., targeted MS/MS, biosensors) for detecting and quantifying aSyn PTMs and pave the way for decoding aSyn PTMs in health and disease. Furthermore, the identification of PTMs that are specific or more abundant in specific types of synucleinopathies could lead to the development of disease-specific biomarkers and diagnostics. Beyond their important use in biomarker discovery and validation, these tools will also help shed new light on the pathophysiology of this protein and consequently offer a better understanding of the molecular mechanisms underpinning synucleinopathies.

### Future directions

To address the aforementioned limitations and challenges, it is essential to (1) standardize protocols for sample collection and handling, aiming to maintain the properties and diversity of aSyn and its modified species; (2) access a large sample set from PD patients and controls as well as patients suffering from other synucleinopathies to conduct biomarker-based studies that reliably assess patient-to-patient variability; (3) standardize selected cohorts regarding disease diagnosis and progression and additional factors (e.g., medical treatment, lifestyle, and comorbidities); (4) harmonize guidelines for the development of novel assays, relying on a panel of well-characterized antibodies and pure and homogeneously modified standards; (5) optimize assays for mapping aSyn PTM patterns at the single-molecule level; and (6) improve the efficiency and sensitivity of MS-based approaches to profile aSyn species using unbiased methods and subsequently quantify these species by targeted methods. Many PTMs may exert their effects by acting on later stages of aSyn aggregation and pathological progression^90^. Hence, developing new approaches to investigate the role of post-aggregation PTMs in regulating aSyn aggregation, pathology spreading and toxicity is essential. In the context of biomarker discovery and validation, future studies should focus on measuring the levels of modified aggregated proteins. Collectively, this will facilitate the development of novel assays to simultaneously measure and quantify the ratios of various aSyn species (e.g., total, phosphorylated, nitrated, aggregated, or modified aggregated species).

The detection of monomeric and aggregated aSyn forms in body fluids (e.g., CSF^44–52^, blood components^53–63^, saliva^64–67^ and tears^68,69^) as well as in peripheral tissues^36,70–72^ presents unique opportunities for the identification and validation of novel disease-relevant aSyn-based markers. However, increasing evidence suggests that it is unlikely that measuring a single aSyn species or total aSyn on its own will provide a sensitive diagnostic biomarker for early disease detection and monitoring of progression.

We propose that the use of a combination of biochemical and structural aSyn biomarkers and NDDs biomarkers is likely to yield a better performance diagnostic for early detection, patient stratification and monitoring of disease progression, thus paving the way for more personalized therapies for the management and treatment of PD and other synucleinopathies. Thanks to recent advances in the detection and amplification of aSyn aggregates in biological fluids and peripheral tissues as well as cryogenic electron microscopy (cryo-EM) in enabling near-atomic-level structural insight into aSyn brain pathology, we are closer to achieving this goal.

The development of aSyn SAAs (i.e., PMCA and RT-QuIC) has enabled the efficient amplifications of minute amounts of aggregated aSyn in CSF^35,85,86,88^, skin biopsies^36,83^, or colon biopsies^72^, thus facilitating the development of assays that enable the differentiation of PD patients from controls with remarkable specificity and accuracy^36,88^, and potentially differentiate PD from other synucleinopathies^36,88^ and predict disease development.

On the structural side, our understanding of the structural properties of aSyn aggregates was initially primarily based on solid-state nuclear magnetic resonance studies performed on aSyn oligomers and fibrils^220,221^. Recently, thanks to advancements in cryo-EM, near-atomic models of aSyn fibrils were obtained from in vitro preparations^28,222–225^ as well as postmortem brain patients with MSA^17,226^. The resulting cryo-EM structures revealed the folding landscape of the aSyn monomers into different polymorphs. To date, most of the structures have been derived from in vitro preparations due to the ease of sample preparation for cryo-EM imaging.

Interestingly, aSyn fibrils derived from the brains of patients with MSA and DLB demonstrated that they have different morphological and structural properties, supporting the idea of disease-specific structure classification of synucleinopathies^227^. Strikingly, the cryo-EM structure of MSA fibrils from the brain^227^ is dramatically different from all the structures of aSyn obtained from different preparations of recombinant aSyn fibrils. This underscores the critical importance of establishing that the tools and assays we use to detect or quantify aSyn aggregates are able to detect native (brain-derived) aggregates, in addition to other types of aSyn fibril structures.

Greater success has been achieved in solving amyloid fibrils from other NDDs, such as AD^228,229^ and ALS^230^. We believe that it is only a matter of time before the structure of aSyn aggregates from different synucleinopathies will be solved. Although several groups have shown progress toward amplifying brain and peripheral-tissue-derived aSyn aggregates using the aSyn SAAs, it remains unclear whether the in vitro amplified structure faithfully reproduces the structural properties of the native aggregates. However, we predict that we will soon have access to methods that allow us to faithfully replicate and amplify the structure of native aSyn aggregates in vitro. These advances will pave the way for a new structure-based classification of synucleinopathies as was recently achieved for tauopathies^231^. Achieving this goal could enable the differentiation of proteinopathies by targeting the different polymorphs and the development of disease-specific structure-based diagnostics and therapeutics.

**Fig. 9:**
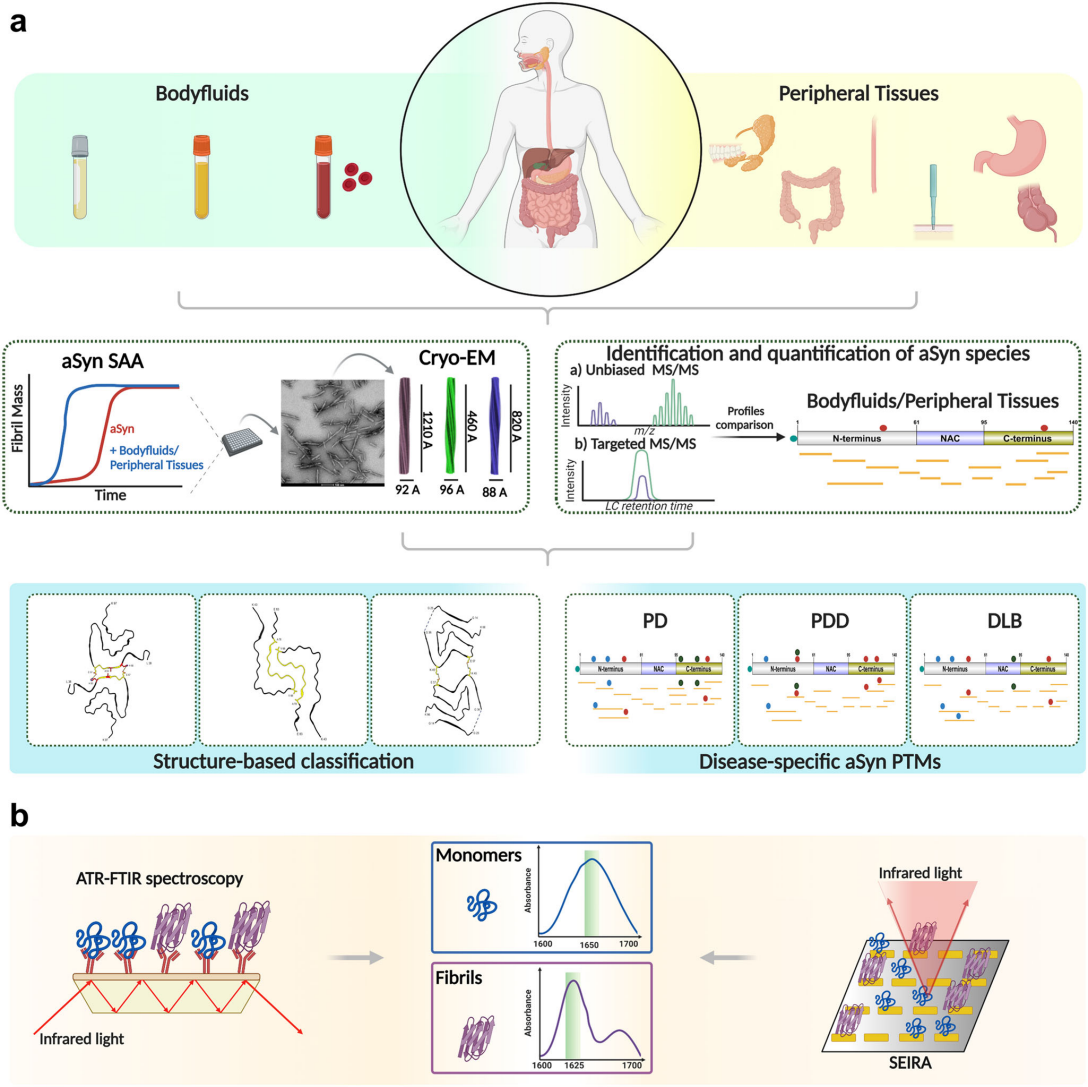
Molecular and structural biomarkers of synucleinopathies. **a** The combination of (1) amplification and detection of minute amounts of aggregated aSyn in biological samples (i.e., aSyn SAA) coupled with cryo-EM and (2) identification and quantification of aSyn species by MS/MS can lead to the discovery and validation of novel biomarkers, relying on structure-based classification and disease-specific aSyn PTMs. Together, these approaches can open new avenues to enable differentiating PD patients from controls and from patients with other synucleinopathies. **b** Schematic depictions illustrating how attenuated total reflection-Fourier transform infrared spectroscopy (ATR-FTIR) and surface-enhanced infrared absorption spectroscopy (SEIRA) could be used as complementary approaches in this workflow for high-throughput structural analysis of biological samples, allowing molecular-level differentiation of a monolayer of aSyn monomers and fibrils. Created with BioRender.com and the cryo-EM structures of the aSyn fibrils depicted in the figure are derived from different aSyn recombinant proteins.

To fully realize the potential of these new advances and develop more reliable biochemical and structural aSyn biomarkers, it is crucial to first map the biochemical and structural diversity of aSyn in biological fluids (CSF and plasma), peripheral tissues and postmortem brain tissues. Quantitative proteomics (MS methods) can be used to determine the distribution of aSyn species defined by different PTMs, as well as the aSyn proteome profile associated with each sample type. A combination of aSyn SAA and cryo-EM approaches can also be used to map the polymorphism and structural diversity of aSyn aggregates (Fig. 9a). For the first time, these methods will allow researchers to ascertain the relationship between the distribution of aSyn species in the brain and CSF and whether the CSF provides a window to aSyn pathology in the brain. Similarly, these studies will also enable the first comparative studies to identify the similarities or differences between aSyn pathology in the brain and peripheral tissues.

We envision a future in which samples from various peripheral tissues and body fluids will be collected at different time points over a follow-up period from patients with different synucleinopathies. These samples will be simultaneously analyzed by (1) aSyn SAA to quantify the levels of aSyn aggregates and perhaps obtain the signature aggregation kinetic profile of aSyn and different amyloid-forming proteins (Tau and TDP-43) in the sample; (2) detection of total aSyn forms using assays that capture the diversity of aSyn species; and (3) detection and quantification of a panel of 4–8 disease-associated posttranslationally modified forms of aSyn using immunoassays or MS-based approaches. The low throughput of cryo-EM precludes its inclusion as a diagnostic tool. However, we anticipate that the availability of a large number of structures from native aSyn fibrils and aggregates will pave the way for simplified high-throughput methods for profiling structural diversity in biological samples. This can be achieved by assembling panels of antibodies targeting different sequences and conformations, which would enable rapid indirect profiling of aSyn fibril structure and polymorphism. Furthermore, the mid-infrared spectroscopy technique could be used as a complementary approach in this workflow, as it is a fast, chemically specific, label-free and nondestructive method capable of identifying secondary structures by leveraging the infrared absorption fingerprints of proteins^232^ (Fig. 9b). To overcome the inherent limitations of classical bulk infrared sensing (low sensitivity and the overlap in absorbance between water and proteins), its derivatives, such as attenuated total reflection-Fourier transform infrared spectroscopy (ATR-FTIR) and surface-enhanced infrared absorption spectroscopy (SEIRA), have been developed to serve as potential structural biomarker sensors by differentiating pathological beta-sheet-enriched neurodegenerative markers from intrinsically disordered/α-helix-enriched healthy monomers. For example, an immunological IR sensor based on the ATR-FTIR technique was developed for the early detection of AD through measuring the overall secondary structure distribution of amyloid-beta in body fluids such as blood and CSF^233,234^. SEIRA is still in its infancy but has already demonstrated its potential to perform conformational differentiation at the molecular level in a monolayer of aSyn fibrils and monomers^235^ and identify structural changes in the protein monolayer at high resolution in real time^236^. The identification of the aSyn proteoforms that are specific to each synucleinopathy (e.g., PD, PDD, DLB) could also enable the development of complementary assays that target specific species or PTM signatures, which would further improve the fingerprinting of biological samples.

In summary, this workflow could enable comprehensive quantitative biochemical and structural profiling of disease-specific aSyn species. When integrated into a point-of-care platform, it could enable early diagnosis and prognosis, patient stratification and guide clinical decisions.

### Other neurodegeneration-related biomarkers for synucleinopathies

Increasing evidence supports the hypothesis that PD and most likely other synucleinopathies do not represent a single entity diseases, but are rather heterogeneous and should be categorized into different subtypes based on the main underlying molecular mechanisms associated with each subtype. In addition, there is a consensus today that PD is characterized by the presence of multiple pathological aggregates, including Tau, amyloid-beta and TDP-43 aggregates^237,238^, which may contribute to the clinical heterogeneity of PD and other NDDs^239,240^. Although some studies have shown a correlation between the presence of multiple pathologies and diseases progression or symptomology^241^, the extent to which the levels of the different copathologies vary during disease progression or between the different synucleinopathies remains a subject of active investigation. The relative contributions of aSyn loss and gain of toxic mechanisms to the development and progression of the different disease subtypes remain unknown. Therefore, it is likely that relying solely on aSyn biomarkers may not be sufficient for differentiating between the different subtypes or monitoring the disease progression for some of PD subtypes. Therefore, we agree with previous recommendations calling on expanding the range of biomarkers to include clinical markers, NDDs biomarkers and biomarkers of biological pathways that have emerged as key drivers of disease development and progression^242^.

**Fig. 10:**
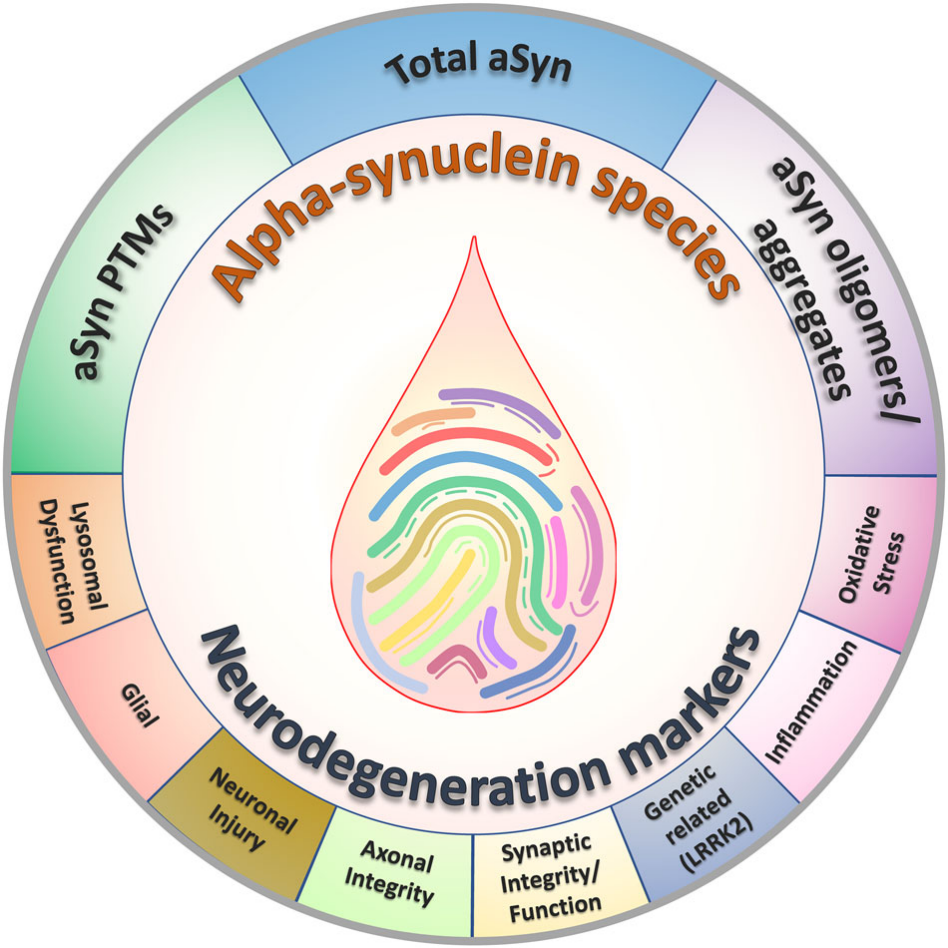
Schematic illustration of biomarker fingerprinting: a multimarker approach including the diversity of aSyn species with other neurodegenerative biomarkers. Partially created with BioRender.com.

This includes biomarkers linked to neuronal injury; axonal integrity and glia (e.g., Tau and phosphorylated Tau, neurofilament light chain (NFL), glial fibrillary acidic protein, vilip-1, YKL-40, TREM2); synaptic integrity/function (e.g., granins, neurotransmitter metabolites, SCG2, PDYN, synaptobrevin); LRRK2-related variables (total and phospho-LRRK2, phospho-RAB); GBA-related variables (Lamp-1, Lamp-2); amyloid-beta; or proteins related to oxidative stress, inflammation, energy failure and the extracellular matrix, such as DJ-1, neurosin, neprilysin, and complement^9,131,243^. For example, CSF levels of the axonal degeneration biomarker NFL may help to differentiate MSA, PSP, and CBD from PD, since its levels were found to be increased in the CSF in the rare synucleinopathy groups compared to PD^131^. Other promising biomarkers linked to synapse neurodegeneration, i.e., VGF, SCG2, and PDYN were found to be reduced in PD and DLB but also correlated with cognitive measurements^243^. For more information on the assessment of the performance of these neurodegeneration biomarkers and their characterization as biomarkers for different synucleinopathy cohorts, please refer to refs. ^9,243^. Although some of these biomarkers have been linked to different NDDs^243^, we predict that the panel of biomarkers encompassing the quantification of different aSyn species with some of the abovementioned neurodegenerative markers could pave the way for more reliable methods for early diagnosis, patient stratification, monitoring disease progression and evaluating target engagement in clinical trials (Fig. 10). These advances would facilitate the development of targeted and potentially personalized therapeutic interventions or disease-modifying therapies for PD and other synucleinopathies.
